# Chemical hybridizing agent SQ-1-induced male sterility in *Triticum aestivum* L.: a comparative analysis of the anther proteome

**DOI:** 10.1186/s12870-017-1225-x

**Published:** 2018-01-05

**Authors:** Hongzhan Liu, Gaisheng Zhang, Junsheng Wang, Jingjing Li, Yulong Song, Lin Qiao, Na Niu, Junwei Wang, Shoucai Ma, Lili Li

**Affiliations:** 1 National Yangling Agricultural Biotechnology & Breeding Center / Yangling Branch of State Wheat Improvement Centre / Wheat Breeding Engineering Research Center, Ministry of Education /Key Laboratory of Crop Heterosis of Shaanxi Province, Northwest A&F University, Yangling, Shaanxi 712100 China; 2 College of Life Science and Agronomy, Zhoukou Normal University, Zhoukou, Henan China

**Keywords:** Male sterility, Proteomics, Protein network, Enzyme activities, Wheat

## Abstract

**Background:**

Heterosis is widely used to increase the yield of many crops. However, as wheat is a self-pollinating crop, hybrid breeding is not so successful in this organism. Even though male sterility induced by chemical hybridizing agents is an important aspect of crossbreeding, the mechanisms by which these agents induce male sterility in wheat is not well understood.

**Results:**

We performed proteomic analyses using the wheat *Triticum aestivum* L.to identify those proteins involved in physiological male sterility (PHYMS) induced by the chemical hybridizing agent CHA SQ-1. A total of 103 differentially expressed proteins were found by 2D–PAGE and subsequently identified by MALDI-TOF/TOF MS/MS. In general, these proteins had obvious functional tendencies implicated in carbohydrate metabolism, oxidative stress and resistance, protein metabolism, photosynthesis, and cytoskeleton and cell structure. In combination with phenotypic, tissue section, and bioinformatics analyses, the identified differentially expressed proteins revealed a complex network behind the regulation of PHYMS and pollen development. Accordingly, we constructed a protein network of male sterility in wheat, drawing relationships between the 103 differentially expressed proteins and their annotated biological pathways. To further validate our proposed protein network, we determined relevant physiological values and performed real-time PCR assays.

**Conclusions:**

Our proteomics based approach has enabled us to identify certain tendencies in PHYMS anthers. Anomalies in carbohydrate metabolism and oxidative stress, together with premature tapetum degradation, may be the cause behind carbohydrate starvation and male sterility in CHA SQ-1 treated plants. Here, we provide important insight into the mechanisms underlying CHA SQ-1-induced male sterility. Our findings have practical implications for the application of hybrid breeding in wheat.

**Electronic supplementary material:**

The online version of this article (10.1186/s12870-017-1225-x) contains supplementary material, which is available to authorized users.

## Background

In hybrid crop breeding, crossing different inbred lines typically results in F1 hybrids that have higher yields than their respective parents. This phenomenon where the hybrid outperforms its parents is known as heterosis [1]. Hybrid crop breeding in maize, rice, sorghum and other species is tremendously successful thanks to cytoplasmic male sterility (CMS) and the three-line system [2]. However, the production of sufficient amounts of hybrid seeds in self-pollinating crops such as wheat is more challenging. Although several types of CMS lines have been bred in wheat (e.g., T-type, K-type and V-type), the three-line system comes with its own limitations. For instance, these lines are more difficult to use due to the lack of fertility restoration sources and the complexity of fertility restoration factors [3]. To combat this issue, chemical hybridizing agents (CHA) have been implemented as an alternative for inducing male sterility in wheat. This method not only enables the production of hybrid seeds of any parental combination, but is also more convenient for promoting heterosis as no maintainer line or pre-breeding is required [4, 5]. However, very limited information is available regarding the proteins and molecular mechanisms behind such CHA-induced sterility.

In wheat, male reproductive processes occur within the anther. Here, diploid sporogenous cells go through meiosis to form haploid microspores that eventually develop into pollen grains [6, 7]. In the case of male sterility, pollen production can be aborted if this elaborate, chronological process becomes disrupted [8]. Previous studies on male sterility in wheat have largely focused on changes in gene expression, enzyme activity and hormone metabolism [9, 10].

Recently, proteomic approaches have been more widely applied to the study of anther development and pollen production. For instance, many proteins specifically expressed in anthers have been detected in rice, Arabidopsis, maize, wolfberry, tomato, and pepper [8, 11]. Furthermore, a large quantity of proteins involved in energy conversion, signal transduction, stress tolerance, the cytoskeleton, transcription, and protein metabolism have been identified in angiosperm pollen [12–14]. In addition, heat shock proteins and β-expansions have been found to be associated with cold temperature stress-induced male sterility in rice [15]. Proteomic analysis of anthers from the male-sterile 7B-1 tomato mutant revealed that the proteasome and the 5B protein - both holding putative roles in tapetum degeneration - are downregulated during the tetrad stage of pollen development [7]. Moreover, a novel male-sterile mutant of *Arabidopsis thaliana* was associated with FLP1, a protein that likely plays a role in the synthesis of sporopollenin, wax, and components of tryphine [16]. Similarly, in wolfberry, the differential expression of numerous proteins, including ATP synthase subunits (energy conversion), the putative callose synthase catalytic subunit (anther development), and various proteases and protease inhibitors, attempt to explain the occurrence of YX-1 male-sterile mutants [8]. In a study on cybrid pummelo, an iTRAQ-based quantitative proteomics approach indicated that the differentially expressed proteins (DEPs) found to be linked with male sterility were mainly involved in carbohydrate and energy metabolism, as well as in protein degradation through the ubiquitin-proteasome pathway [17]. In rapeseed, a 2-DE analysis of CHA-induced male sterility revealed that some of the DEPs were related to tapetum development. These proteins, which were found to be downregulated, might disrupt the normal development of tapetum and microspores. In this way, these structures would be rendered unviable and finally pollen abortion would result in male sterility [18]. Moreover, in a previous study on poly-ubiquitinated proteins in SQ-1-indcued male sterile wheat, we found that male sterility is closely related to the poly-ubiquitination degradation of the sterile plants [19].

Wheat is a critical cereal crop that is cultivated at a global level, supplying nearly 20% of the world’s daily food in the form of important principal grains [3, 20].. As such, it is necessary to understand male sterility in this organism in order to increase the production of hybrid seeds, and thus overall yield. There are three fundamental systems for hybrid seed production with respect to crops: cytoplasmic male sterility (CMS), genic male sterility (GMS), and CHAs [21]. The use of CHAs is a favorable system for inducing male sterility in wheat because it does not require fertility restoration. However, both the large size and polyploidy complexity of the wheat genome act as considerable barriers to genome analyses. Thus, proteome analysis of developing anthers could be a more appropriate method when studying CHA SQ-1-induced male sterility in wheat.

Currently, hybrid wheat is considered as the first choice to increase wheat yield in the near future. It is also a major focus of international competition for the agricultural high-tech and modern seed industries [22]. In the present study, we performed comparative proteomic analysis to identify DEPs in fertile and CHA-induced male sterile anthers at different stages of development. Subsequently, we examined the possible biological functions of these DEPs, and discussed their potential effects on anther development and male sterility.

## Methods

### Plant material and anther collection

In the present study, wheat cultivar (cv. Xinong 1376) was grown in the experimental field of Northwest A&F University in Yangling, Shaanxi Province, P.R. China (34° 16’ N, 108° 4′ E). The following April when the wheat had reached a growth stage of 8.5 according to the Feekes scale (as described in [23]), wheat plants were divided into two groups, with 50 rows per group. While plants from one group were treated with the CHA SQ-1 (5 kg ha^−1^) and named PHYMS-1376, the other plants, which were sprayed with water as a control, were named MF-1376. After ten days, we examined wheat anther morphology and cell structure (light microscope) to determine the developmental stage of the anthers. Finally, both MF-1376 anthers and PHYMS anthers were collected, frozen in liquid nitrogen, and stored at −80 °C until further analysis.

### Histological analysis and phenotypic characterization

The details of bright-field photographs of individual spikelets, flowers and anthers were described previously [19]. We used carbol fuchsin to stain the nuclei of wheat anthers at various stages of development, and examined and photographed the nuclei with a Nikon ECLIPSE E600 optical microscope. Scanning electron microscopy (SEM) of fresh pollen grains and freeze-dried anthers were performed as described by [24]. Anthers in the trinuclear stage of development from both MF-1376 and PHYMS plants were collected from spikelets just prior to anthesis. Pollen grains were stained with 1% iodine-potassium iodide solution (1% KI-I_2_).

### Preparation of paraffin-embedded sections

Anthers were collected at various stages of development, fixed in FAA (50% ethanol, 10% formalin, and 5% acetic acid), and dehydrated using a graded ethanol series [25]. For histological analysis, tissues were then infiltrated with xylene and embedded in Paraplast Plus. Finally, materials were cut into 12-μm-thick sections, stained with 0.5% toluidine blue (Sigma), and photographed using a Nikon ECLIPSE E600 microscope.

### Protein sample preparation and quantitation

Anther proteins were extracted according to the method of trichloroacetic acid (TCA)-acetone procedure as described by Sheoran and Sawhney with minor modifications [26]. The details of operation steps were described previously [19]. The protein concentrations of the final extracts were quantified using the Bio-Rad protein assay reagent (Bio-Rad, USA), and finally stored at −80 °C for 2-DE.

### Gel electrophoresis and data analysis

We performed 2-DE according to established procedures. We loaded 200 μg of each protein sample onto 17-cm Immobiline Drystrips of 4–7 non-linear pH gradients (Bio-Rad, CA, USA), rehydrating them passively with 350 μL of protein solution for 14 h at 20 °C. First-dimension gel electrophoresis was performed on the Protean IEF cell (Bio-Rad, CA, USA) at 20 °C with a 50-mA current limit per strip. We applied the following voltage gradient: 250 V for 1 h, 500 V for 1 h, 1000 V for 1 h, 4000 V for 1 h, and 8000 V until a total of 80,000 VH were reached. Finally, a constant voltage (500 V) was applied for the last 12 h. The focused strips were then equilibrated twice (15 min each) with gentle shaking in a solution containing 0.375 M Tris-HCl (pH 8.8), 6 M urea, 20% (*v*/v) glycerol, and 2% (*w*/*v*) SDS. On top of this, 2% DTT was added in the first equilibration and 2.5% iodoacetamide in the second equilibration. For the second dimension, proteins were separated on 12% SDS-PAGE gels using a PROTEAN II Multi Cell (Bio-Rad, CA, USA). SDS-PAGE was run at 10 mA per gel for 1 h, followed by 20 mA per gel until the bromophenol blue dye reached the base.

Protein spots were visualized by silver staining, and scanned at 300 dpi using a Powerlook 2100XL imaging densitometer (UMAX Technologies, Dallas, TX, USA). Image analysis was performed using the software PDQuest 7.4 (Bio-Rad, CA, USA) according to the manufacturer’s instructions. Quantitative image analysis was conducted to identify those protein spots with reproducible and significant differences in abundance (vol.% > 1.5 fold; *p*-value <0.05).

### In-gel digestion and MALDI-TOF/TOF MS analysis

Selected protein spots were manually cut from the gel and digested using sequencing-grade trypsin. The gel spots were successively destained and dehydrated with 30 mM K_3_Fe(CN)_6_ in 100 mM Na_2_S_2_O_3_. Then, the proteins were reduced with 10 mM DTT in 25 mM NH_4_HCO_3_ at 56 °C for 1 h, and alkylated in the dark at room temperature for 45 min using 55 mM iodoacetamide in 25 mM NH_4_HCO_3_. Finally, gel pieces were thoroughly washed with 25 mM NH_4_HCO_3_ in 50% acetonitrile, dehydrated with 100% acetonitrile, and completely dried in a SpeedVac (Savant, UK) concentrator. Proteins were digested in 5 μL of 2.5–10 ng/μL sequencing-grade trypsin solution (Promega) overnight at 37 °C. The resulting tryptic digests were concentrated and desalted using C_18_ ZipTips (Millipore Corporation, Bedford MA) according to the manufacturer’s instructions. Tryptic peptides were dissolved and analysed as described by [27].

### Bioinformatics analysis

Hierarchical cluster analysis of the DEP spots was performed using the log-transformed data and the Multiple Experiment Viewer 4.9 software. All identified proteins were blasted against the TAIR (The Arabidopsis Information Resource) and *Brachypodium distachyon* (a new model plant from the family gramineae) protein databases and used for constructing a protein–protein interaction (PPI) network. Biological pathway networks were generated with the Cytoscape plug-ins BiNGO and CLUEGO.

### Enzyme activity assays

We measured β-1, 3-Glucanase activity as described by Torres et al. [28], and vacuolar invertase activity as described by Tomlinson et al. [29]. According to the method of Cheng et al. [30], ROS content was determined by measuring the levels of O_2_^−^, H_2_O_2_, and malondialdehyde (MDA), and antioxidant enzyme activity was determined by assaying the superoxide dismutase (SOD), peroxidase (POD) and catalase (CAT) enzymes.

### Quantitative real-time PCR analysis

Total RNA was extracted from PHYMS and MF-1376 anthers at different developmental phases. Primers for quantitative real-time PCR (qRT-PCR) analyses were designed using the primer premier 5.0 software. The specific primers for *Ivr5* (Accession number AF069309) were: 5’-TTCACTGTGCCTGTGCTCG-3′ and 5′-TCCGTCGGATACACCCTC-3′. 18S (Accession number AY049040) was used for RNA normalization. qRT-PCR was performed on a BIORAD CFX96 real-time system. The 25 µL reaction and the cycling parameters were described previously [31]. During the final amplification round, PCR reaction specificity was checked by melting curve analysis (60 to 95 °C in increments of 0.5 °C every 5 s). Each experiment described above was repeated independently three times. The data were analyzed using the 2^-∆∆Ct^ method by Livak and Schmittgen [32].

## Results

### Phenotypic differences between MF-1376 and PHYMS wheat

**Fig. 1:**
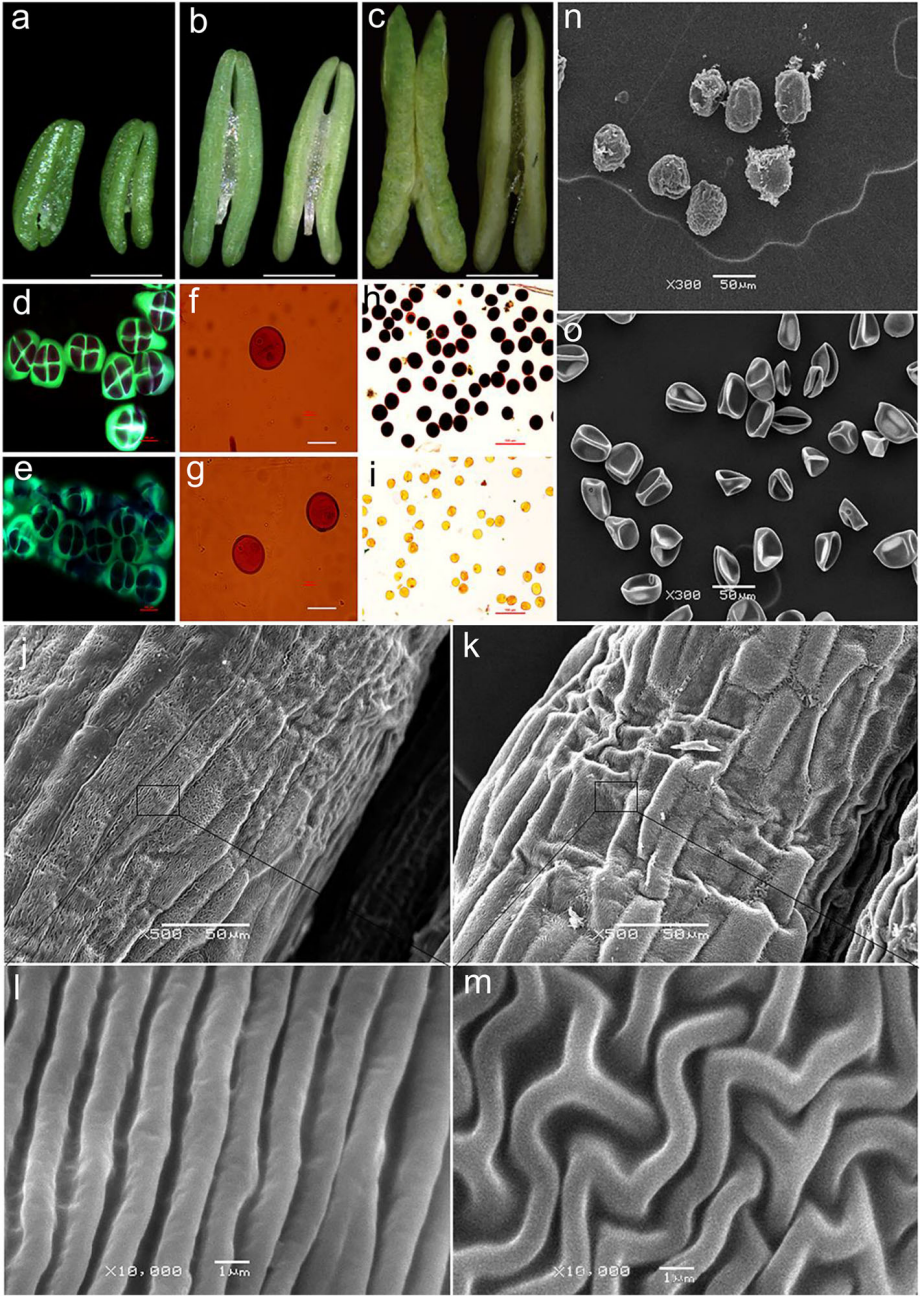
Phenotypic traits of both PHYMS wheat and the corresponding fertile line, MF-1376. **a**-**c** The morphology of MF-1376 (left) and PHYMS (right) anthers at the (**a**) tetrad, (**b**) mononuclear and (**c**) trinuclear stage of development. **d** Callose deposition in MF-1376 anthers at the tetrad stage. **e** Callose deposition in PHYMS anthers at the tetrad stage. **f**-**g** Anther nuclei from both (**f**) MF-1376 and (**g**) PHYMS wheat were stained at the mononuclear stage with carbol fuchsin. **h** Pollen grains from a fertile plant stained with KI-I_2_ solution. **i** Pollen grains from a male-sterile plant stained with KI-I_2_ solution. **j**-**m** SEM analysis of the surface of trinuclear stage anthers from (**j**, **l**) MF-1376 and (**k**, **m**) PHYMS wheat. **n**-**o** SEM analysis of pollen grains from (**n**) MF-1376 and (**o**) PHYMS wheat. Bars = 2 mm in (**a**-**c**), 100 μm in (**d**-**i**), 50 μm in (**j**-**k**, **n**-**o**), and 1 μm in (**l**-**m**)

We compared the phenotype of MF-1376 and PHYMS anthers at all three stages of development (Fig. 1a-c). Although we found no marked difference between MF-1376 and PHYMS anthers at the tetrad and mononuclear stages, MF-1376 anthers, but not PHYMS anthers showed anther dehiscence at the trinuclear stage (Fig. 1c). Typically, staining anthers with carbol fuchsin-aniline blue is a convenient way to observe the behavior of the callose wall at the tetrad stage. This fluorescence method yields vivid and colorful photos, where the cytoplasm appears red, the chromosomes carmine, and the callose dark green. We found that while the callose of MF-1376 anthers at the tetrad stage stained a bright color and showed a thick deposition, the callose of PHYMS anthers was dull in color and exhibited an abnormal deposition (Fig. 1d and e). We did not detect any visible differences at the mononuclear stage between the microspores of MF-1376 and PHYMS anthers (Fig. 1f and g). Staining of pollen grains with KI-I_2_ indicated that the pollen grains of MF-1376 exhibited pronounced starch accumulation (Fig. 1h), whereas those of PHYMS were almost entirely starch deprived (Fig. 1i). Moreover, we used SEM to further analyze trinuclear stage anthers for ultrastructural characteristics. We found that the exterior of MF-1376 anthers possessed a well-formed cuticle (Fig. 1j and l), while in contrast, the outer surface of PHYMS anthers was fairly disorganized (Fig. 1k and m). In addition, MF-1376 pollen grains showed a smooth and particulate exine pattern, and a nearly round shape (Fig. 1n), whereas PHYMS pollen grains were severely malformed (Fig. 1o). These observations agree with the KI-I_2_ staining, and indicate that both the anther wall and pollen grains of PHYMS plants had defective development. Moreover, the relative male sterility rate in the PHYMS wheat was as high as 99.68%, with a 96.35% seed-setting rate of artificial pollination and no pistil damage.

### Characterization of anther development at the microscopic level

**Fig. 2:**
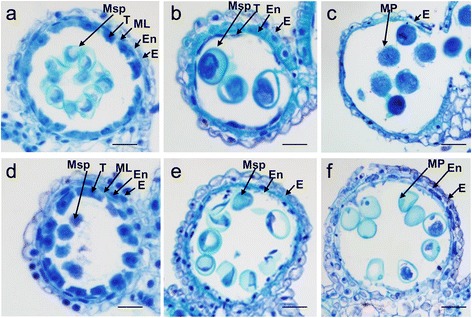
Comparison between transections of fertile and male-sterile anthers.**a**-**c** Cross sections of a MF-1376 locule at the (**a**) tetrad, (**b**) mononuclear and (**c**) trinuclear stage of development. **d**-**f** Cross sections of a PHYMS locule at the (**d**) tetrad, (**e**) mononuclear and (**f**) trinuclear stage of development. Cross sections were stained with 0.25% toluidine blue. E, epidermis; En, endothecium; ML, middle layer; Msp, microspore; MP, mature pollen; T, tapetum. Bars =12 μm

To more accurately determine the timing of developmental defects in PHYMS anthers, we inspected the anthers at the microscopic level. Anthers of both MF-1376 and PHYMS plants have walls comprised of four layers, which are from the surface to the interior, the epidermis, the endothecium, the middle layer, and the tapetum (Fig. 2a and d). These four somatic layers surround small cavities known as locules, which harbor at their center the plants’ microspores. At the tetrad stage, we found no significant differences between the MF-1376 and PHYMS anthers with respect to the four somatic layers. However, at this stage a significant difference can be seen with respect to the microspores, with the periphery of the MF-1376 microspores having an extra layer that is not seen around the PHYMS microspores. At the mononuclear stage, the anther walls (including both the tapetum and the middle layers) of MF-1376 plants showed normal degeneration (Fig. 2b), whereas the tapetum layer of PHYMS anthers had almost entirely degraded (Fig. 2e). In addition, the endothecia in MF-1376 anthers were thicker than in PHYMS anthers, and at the mononuclear stage the MF-1376 microspores had more starch accumulation than the PHYMS microspores (Fig. 2b and e). Furthermore, unlike the mature pollen grains found in MF-1376 trinuclear stage anthers (Fig. 2c), pollen in the PHYMS anther locules were nearly collapsed and showed little starch accumulation (Fig. 2f). At the trinuclear stage, the layers of the anther wall were further degenerated in MF-1376 plants and in fact, only the epidermis layer remained intact (Fig. 2c). This suggests that during this stage, lipophilic materials (cutin and wax) diffuse to the surface of the anther cell wall. In contrast, the layers of the PHYMS anther wall appeared less degraded. For instance, we observed a clear endothecium, thick epidermis, and defined cellular structures (Fig. 2f). This implies that decreased amounts of lipophilic materials had been deposited or transferred to the endothecium and outer epidermal cell wall during this stage.

### 2D–PAGE analysis of anther proteomes

Anther proteins from both MF-1376 and PHYMS wheat cultivars were extracted (three biological replicates) and independently separated by 2-D PAGE. In both the MF-1376 and PHYMS groups, we reproducibly detected more than 800 protein spots on silver-stained gels. Of these, 466 were found on all gels. We identified a total of 191 differential spots after filtering for proteins exhibiting a > 1.5-fold increase or decrease in expression (*p* < 0.05; one-way ANOVA).

**Fig. 3:**
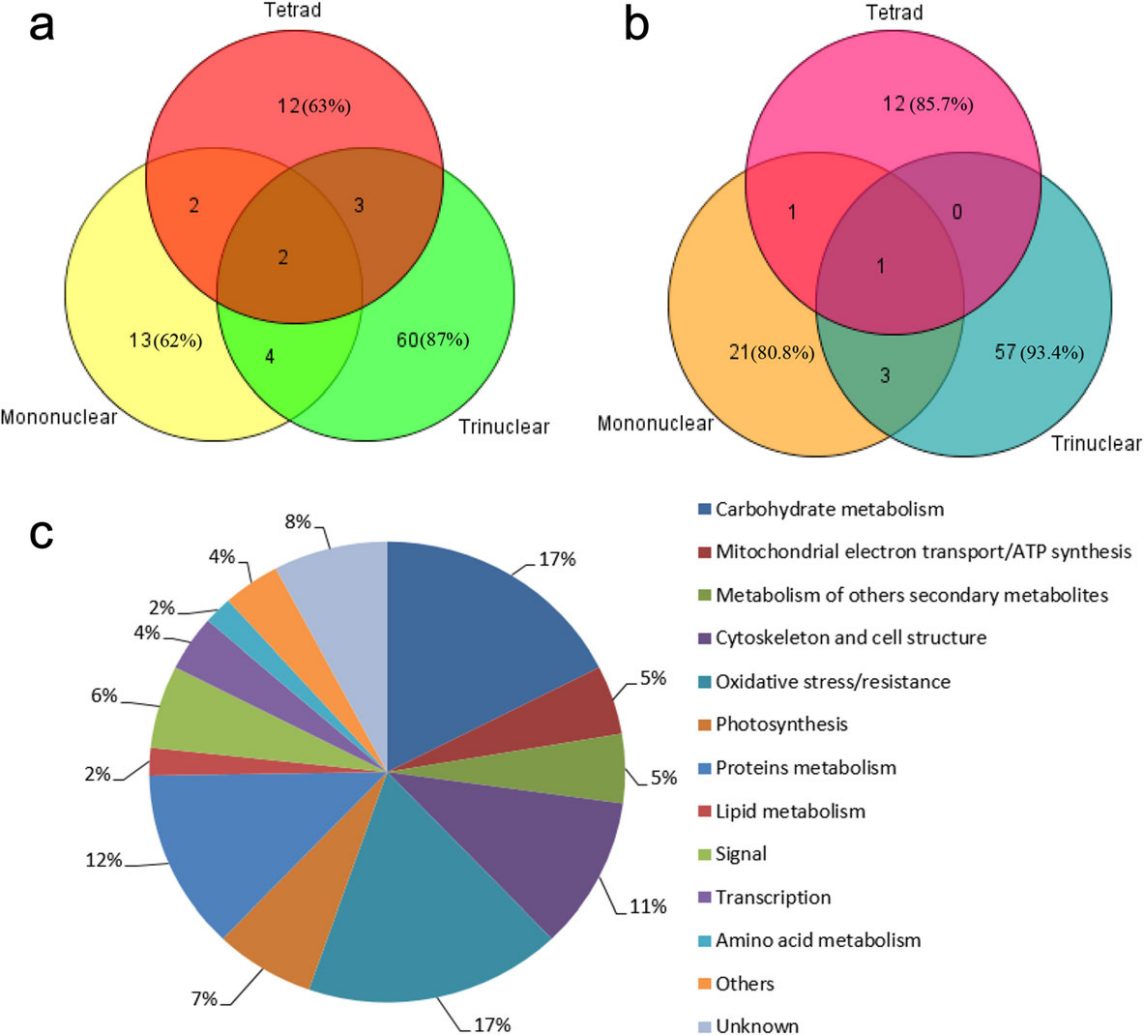
Stage-dependent distribution of DEPs and their classification into functional categories. **a** Venn diagram representing the number of proteins upregulated in PHYMS anthers (compared to MF-1376 anthers) at each of the three stages of development. **b** Venn diagram representing the number of proteins downregulated in PHYMS anthers (compared to MF-1376 anthers) at each of the three stages of development. **c** Functional classification and distribution of all 103 identified proteins based on sequence homology analysis as listed in Additional file 2: Table S1. Thirteen groups of protein species were categorized based on the putative functions of homologous proteins. The percentage of proteins in each group is indicated. Different colors represent different functional groups

Due to the sensitivity and reproducibility of 2-DE technology, a 1.5-fold expression difference was employed as the threshold limit and three replicates were performed to reduce the number of potential false positives. Six representative 2D gel maps separated with IPG 4–7 strips are shown in Additional file 1: Figure S1, with the spots used for mass spectrometry analysis indicated and numbered. We used Boolean analysis and Venn diagrams to illustrate the number of DEPs and their overlap between the different stages of anther development (Fig. 3a and b). A total of 96 different proteins showed at least a 1.5-fold (*p* < 0.05) increase in abundance in PHYMS anthers compared to in MF-1376 anthers. This included 19, 21 and 69 proteins at the tetrad, mononuclear, and trinuclear stage, respectively, with 2 of them (spot 1 and spot 9) being more abundant in all three stages, and 9 of them in two of the three stages (spot 2 and spot 3 in the tetrad and mononuclear stages; spots 4, 5 and 10 in the tetrad and trinuclear stages; and spots 6, 7, 8 and 11 in the mononuclear and trinuclear stages). The remaining 85 proteins were upregulated in only one stage: 12 proteins in the tetrad stage, 13 in the mononuclear stage, and 60 in the trinuclear stage (Fig. 3a). Among the 95 downregulated proteins, only one protein (spot 13) was downregulated in all three stages, and 4 proteins in any two of the stages (spot 12 in the tetrad and mononuclear stages; no protein common to the tetrad and trinuclear stages; and spots 14,15 and 16 in the mononuclear and trinuclear stages). The remaining 90 proteins were downregulated in only one stage: 12 proteins in the tetrad stage, 21 in the mononuclear stage, and 57 in the trinuclear stage (Fig. 3b).

### Protein identification

To identify the up- and downregulated proteins, we performed MALDI-TOF/TOF MS and MALDI-TOF/TOF MS/MS analyses on the excised spots. From this, we successfully identified 103 protein spots by searching against the NCBInr and UniProt databases (Additional file 2: Table S1). Enlarged regions of the 2D–PAGE gels for several of the excised spots are shown with corresponding 3D views (Additional file 3: Figure S2). The identified proteins were classified into 13 major categories according to their putative physiological functions: carbon metabolism (17%), mitochondrial electron transport/ATP synthesis (5%), metabolism of other secondary metabolites (5%), cytoskeleton and cell structure (11%), oxidative stress/resistance (17%), photosynthesis (7%), proteins metabolism (12%), lipid metabolism (2%), cell signaling (6%), transcription (4%), amino acid metabolism (2%), other functions (4%), and unknown proteins (8%) (Fig. 3c). To further visualize protein expression patterns, we constructed heat maps with hierarchical clustering of the DEPs according to their percent (%) volume values. We created a dynamic expression profile for the DEPs identified at each of the three stages, using MF-1376 protein values from every anther stage as benchmark values by K-means clustering. When compared to the tetrad, mononuclear and trinuclear stages of MF-1376 (benchmark values), we found 10 distinct expression patterns for the DEPs. And these patterns contain 10 DEPs, 27 DEPs, 4 DEPs, 18 DEPs, 15 DEPs, 4 DEPs, 13 DEPs, 3 DEPs, 5 DEPs and 4 DEPs, respectively (Additional file 4: Figure S3).

### Bioinformatics-based PPI network analysis of key DEPs involved in wheat male sterility

**Fig. 4:**
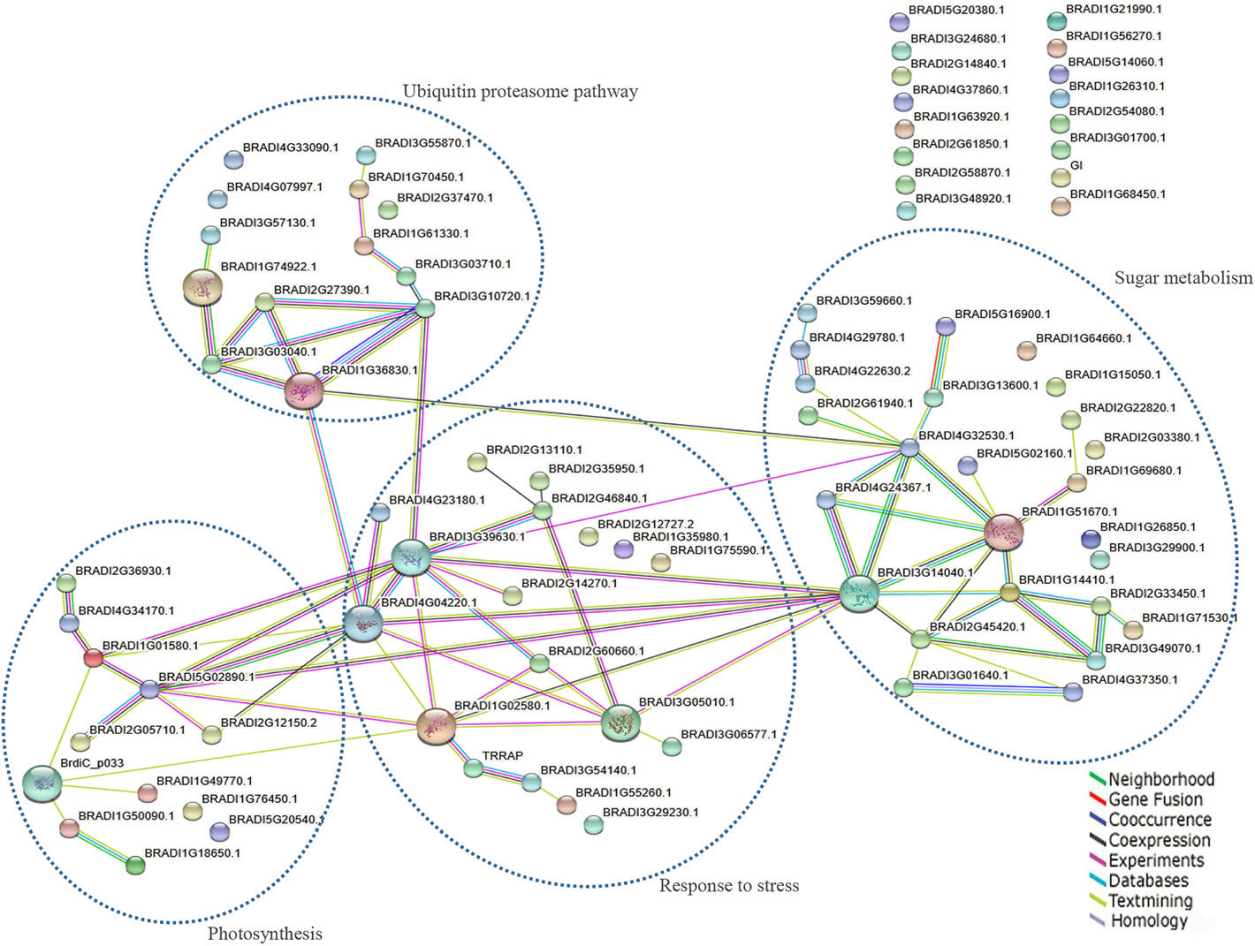
Protein interaction network analysis using STRING 10.0. DEPs were mapped to *Brachypodium distachyon* homologs by searching the STRING 10.0 database (http://string-db.org) with a confidence cutoff of 0.4. Colored lines between the proteins indicate the type of interaction evidence. Details of all the protein nodes are listed in Additional file 5: Table S2a

To explore the relationship between all the identified DEPs, we created a PPI network by blasting the 103 DEPs against both the TAIR and *Brachypodium distachyon* protein databases (Additional file 5: Table S2a and b). The PPI network based on the *Brachypodium distachyon* homologs revealed four important functional categories principally involved in sugar metabolism, the stress response, photosynthesis, and the ubiquitin proteasome pathway (Fig. 4). These four functional categories were not fully separated, but rather formed an interconnected network regulating anther infertility. The sugar metabolism and stress response (defense/detoxification) groups included a larger number of members, with interactions being concentrated on glucose-6-phosphate isomerase-like (BRADI4G32530.1)/glyceraldehyde-3-phosphate dehydrogenase (BRADI3G14040.1) and heat shock protein (BRADI3G39630.1; BRADI4G04220.1), respectively. With respect to the photosynthesis and ubiquitin proteasome pathway groups, the RuBisCO large subunit-binding protein subunit alpha (BRADI5G02890.1) and the 26S protease regulatory subunit 8 homolog A-like (BRADI1G36830.1)/ 26S protease regulatory subunit 6B homolog (BRADI3G10720.1) were the most important nodes, respectively. In addition to the aforementioned functional categories, we also identified another two functional categories from the TAIR-based PPI network: cytoskeleton related, and male sterility/anther wall related (Additional file 6: Figure S4). The specific protein names in the two PPI networks are shown in Additional file 5: Table S2a and b.

To further identify statistically over- or under-represented categories of cellular components, molecular functions and biological pathways related to male sterility induced by CHA SQ-1, we applied the Biological Networks Gene Ontology tool (BiNGO) to the DEPs (Additional file 7: Figure S5 and Additional file 8: Table S3). The results reveal that the most highly enriched cellular components are the plasma membrane (*P* = 1.17E–4), the cytoplasm (*P* = 3.12E–12), and the cell wall (*P* = 3.56E–10). Here, the term ¨cytoplasm¨ includes the plastid (*P* = 5.07E–7), cytosol (*P* = 1.3E–15) and vacuole (*P* = 2.41E–5). Meanwhile, the “molecular functions” GO terms that were enriched in DEPs included catalytic activity (*P* = 6.71E–12) and binding (*P* = 1.07E–6). Biological process analysis by BiNGO showed that the DEPs were more highly enriched in metabolic processes (*P* = 1.28E–9), cellular processes (P = 6.71E–12), the response to abiotic processes (*P* = 5.44E–7), and the response to stress (*P* = 3.14E–6). More specifically, within the metabolic processes category, generation of precursor metabolites and energy (*P* = 3.47E–10), carbohydrate metabolism (*P* = 5.98E–9), and catabolic processes (*P* = 1.11E–5) were significantly over-represented.

**Fig. 5:**
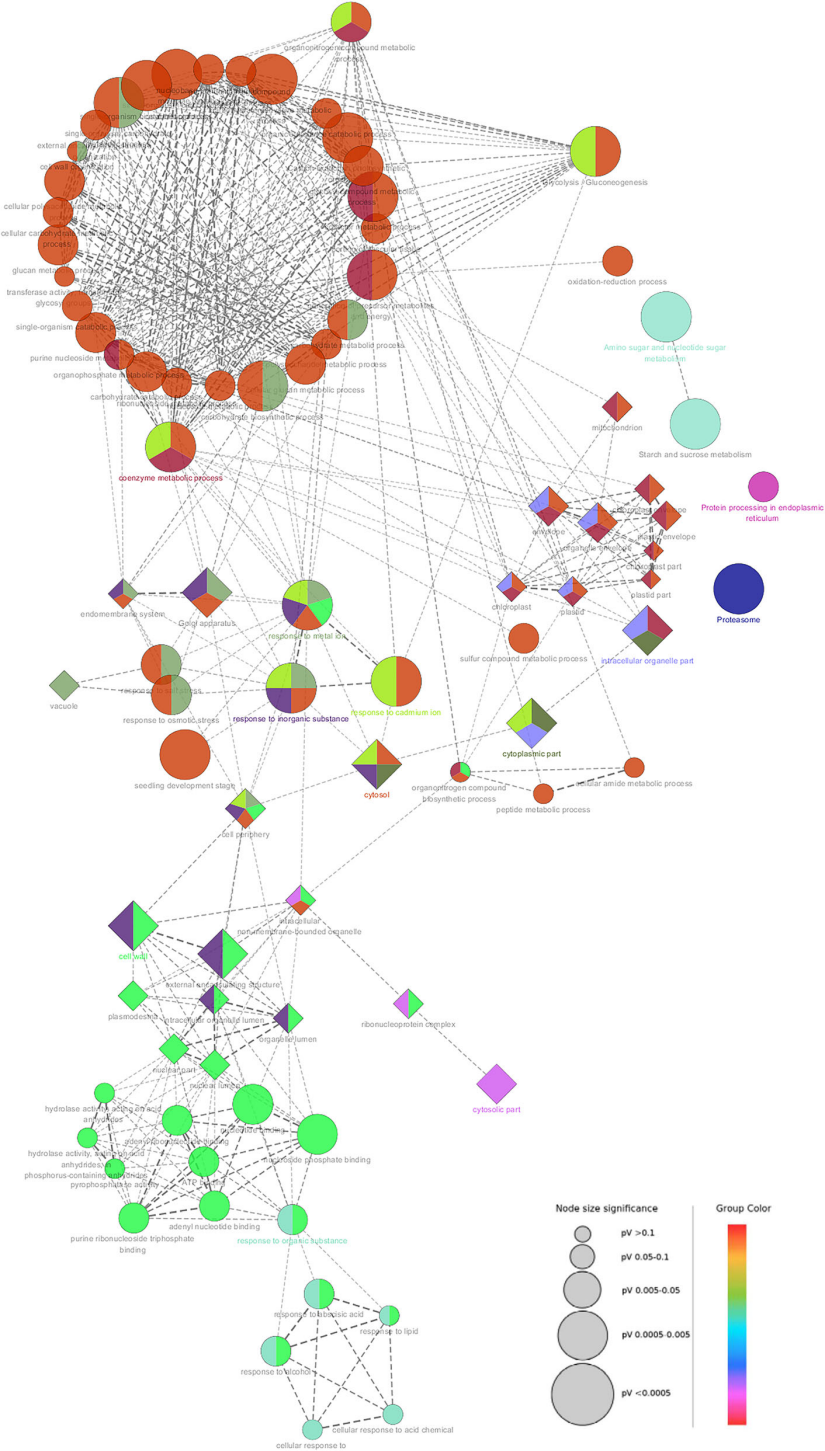
A functionally grouped network of terms and pathways as generated by ClueGO. GO and KEGG terms are represented as round and square nodes, respectively, and linked to each other based on their kappa score levels (≥0.4). The size of the node represents the significance of the term enrichment. Groups are color-coded as indicated on the figure. Details of the entire grouped network are listed in Additional file 9: Dataset S1

In order to create and visualize a functionally grouped network of terms/pathways based on molecular functions, biological processes, cellular components, plant structure and KEGG pathways, we generated a KEGG-GO map using the Cytoscape plug-in ClueGO [33, 34]. The resultant network has 87 terms connected by 466 edges with the kappa scores, and confirmed that the majority of up- and downregulated DEPs are involved in glycometabolism, oxidation-reduction reaction, the response to inorganic substances (metal ions and cadmium ions), chloroplast organization, the vacuole, the cell wall, the proteasome, the response to abscisic acid (ABA), and lipid and alcohol metabolism (Fig.5 and Additional file 9: Dataset S1). These results indicate that male-sterile plants not only suffer from ROS stress and developmental stress, but that their energy supply pathway also has a significant problem. Interestingly, these network clusters could shed light on different roles for the DEPs. Whether directly or indirectly, these DEPs are involved in a wide range of biological pathways that play a role in the process of male sterility in wheat.

### Enzyme activity and gene expression of selected proteins

**Fig. 6:**
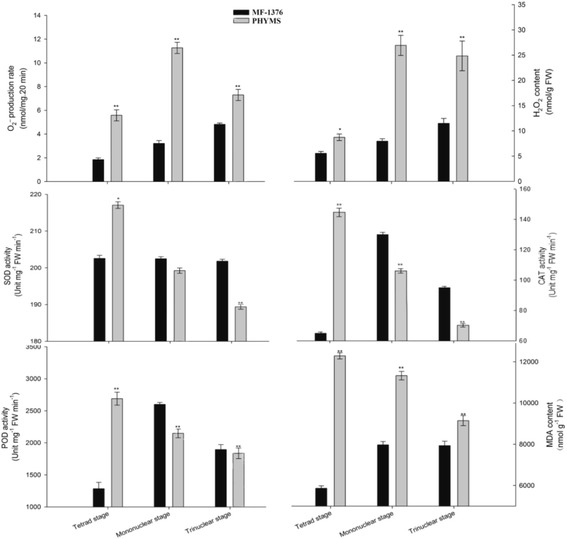
ROS accumulation and SOD, CAT and POD enzymatic activities in developing anthers. The superoxide anion, hydrogen peroxide and MDA content in MF-1376 and PHYMS anthers was measured at the tetrad, mononuclear and trinuclear stage of development. The enzymatic actives of superoxide dismutase, peroxidase and catalase in MF-1376 and PHYMS anthers were also measured at each stage. A single sample was used for three independent replicates (*n* = 3). *, ** = significantly different from the MF-1376 control at *p* < 0.05 and *p* < 0.01, respectively

We identified the putative glutathione S-transferase GSTF1, SOD, and the putative In2.1 protein as DEPs involved in oxidative-redox stress. These three proteins are activated under antioxidant environments in order to scavenge ROS. To confirm the change in expression of these proteins and to correlate this change with enzyme activity, we determined both ROS levels and antioxidant activity. We found that the rate of O_2_^−^ production in PHYMS anthers is significantly higher than in MF-1376 anthers in the tetrad, mononuclear, and trinuclear stages. As excess O_2_^−^ is catalyzed to form H_2_O_2_, we were not surprised to find that PHYMS anthers also possessed a greater H_2_O_2_ content in all three stages. ROS scavenging is known to be dependent on antioxidant enzymes, however, the activities of SOD, CAT and POD in PHYMS anthers were significantly lower than in MF-1376 anthers in both the mononuclear and trinuclear stage. Furthermore, under excess ROS conditions, polyunsaturated lipids are typically degraded to form MDA. We found that MDA levels were significantly higher in PHYMS anthers than in MF-1376 anthers (Fig. 6). Together, these results indicate that PHYMS anthers contain abnormal ROS levels, which might be to affect the tapetal cells, photosynthesis and carbon metabolism, and cell wall biosynthesis and degradation [35, 36]. We determined spots 15 and 16 to correspond to endo-β-1, 3-glucanase and vacuolar invertase 1. We found that the activity of β-1, 3-glucanase in PHYMS anthers was significantly lower than in MF-1376 anthers from the tetrad stage to the trinuclear stage (Additional file 10: Figure S6a). In contrast, while vacuolar invertase activity showed no difference between the MF-1376 and PHYMS anthers in the tetrad stage, in the mononuclear and trinuclear stages the MF-1376 anthers exhibited significantly more vacuolar invertase activity (Additional file 10: Figure S6b). Accordingly, the expression level of *Ivr5* showed a significant difference between the two types of anthers in the mononuclear and trinuclear stages, especially in the trinuclear stage, where the level of *Ivr5* mRNA was nearly 2.85 times higher in MF-1376 anthers than in PHYMS anthers (Additional file 10: Figure S6c). This correlates well with the 2-D PAGE and enzyme activity results.

## Discussion

Although male sterility offers great potential in terms of wheat heterosis, to the best of our knowledge no study has characterized CHA-induced changes in protein expression in wheat anthers at the proteomic level. As such, we have conducted a comprehensive proteomics analysis between MF-1376 and PHYMS anthers in the tetrad, mononuclear and trinuclear stages of anther development in order to gain a better understanding of the mechanisms behind CHA-induced wheat male sterility. We identified a total of 103 DEPs with functions predominantly in carbohydrate metabolism and oxidative-redox stress. As oxidative-redox stress proteins are activated under oxidative stress conditions to scavenge excess ROS, we decided to analyze both the ROS levels and antioxidant activity. Moreover, the potential roles and mRNA expression levels of a few of the other DEPs (e.g., β-1, 3-glucanase and vacuolar invertase) are discussed below.

The putative In2.1 protein, the putative glutathione S-transferase GSTF1, and SOD are all known to be involved in scavenging ROS [37]. The putative In2.1 protein, which has been identified as a homolog of GSTF, was only expressed in PHYMS anthers (Additional file 3: Figure S2, In 2.1). In both crops and weeds, GSTF and or In2.1 detoxify electrophilic herbicides by catalyzing their conjugation with glutathione. Apart from their functions in herbicide detoxification, stress signaling, and apoptosis regulation, GSTF and protein In2.1 are glutathione peroxidases that function as ROS scavengers under various stresses [38]. It is possible that spraying an exogenous agent (i.e., a CHA) on the crops in this study might have caused a toxic effect on anther growth and development. This could explain why we found oxidative-redox stress proteins to be more highly expressed in PHYMS anthers than in MF-1376 anthers. ROS are a by-product of aerobic metabolism, and when high levels of ROS are sustained inside the cell, an organism suffers from oxidative stress. This results in the damage of proteins and nucleic acids, lipid peroxidation, and even necrocytosis [39]. The fact that we found a significantly higher rate of O_2_^−^ production, H_2_O_2_ content, and MDA content in PHYMS anthers indicates that they experience a substantial accumulation of ROS. Simultaneously, PHYMS anthers also demonstrated lower activities of the ROS scavenging enzymes SOD, POD, CAT (Fig. 6). According to the 2-D PAGE gel, the expression of SOD was lower in the PHYMS anthers at both the mononuclear and trinuclear stage of development. These results suggest that an accumulation of ROS, together with lower expression levels of oxidative-redox stress proteins and ROS scavenging enzymes, could result in tapetal degeneration and pollen apoptosis. Furthermore, the chronic oxidative stress caused by aberrant increases in ROS levels may be the cause behind abortion of microspores in PHYMS plants.

Carbohydrate metabolism is a fundamental metabolic pathway for biological systems. Its main physiological function is to provide both the carbon and energy sources that are necessary for proper growth (Fig. 5). Six of the identified proteins are involved in carbohydrate metabolism. Cytochrome c oxidase (COX), the last enzyme of the mitochondrial electron transport chain, was strongly upregulated at all three stages in PHYMS anthers (Additional file 3: Figure S2, CCOS). COX is known to be regulated by isozyme expression, allosteric effectors such as the ATP/ADP ratio, and reversible phosphorylation [40]. The fact that we found COX to be upregulated in all stages of PHYMS anthers suggests that it also plays a role in male sterility. Under CHA stress conditions, COX probably facilitates energy generation via the respiratory chain.

Alternative oxidase (AOX), a component of the alternative electron transport chain in the inner mitochondrial membrane of numerous organisms, has the ability to suppress ROS production, the stress response, and signals of programmed cell death [41]. In our study, we found that AOX was more highly expressed in MF-1376 anthers than in PHYMS anthers at both the tetrad and trinuclear stage. Our tissue cross sections revealed that there are significant differences between the microspores of the two plants at the tetrad stage. For instance, only the periphery of the MF-1376 microspores had a packing layer surrounding the spores. These results indicate that the abnormality of microspores at the tetrad stage might be related to the difference in AOX expression between MF-1376 and PHYMS anthers.

The UDP-forming α-1,4-glucan-protein synthase is associated with the formation of cell wall polysaccharides such as hemicellulose and xylose [42]. The higher expression of UDP-forming α-1,4-glucan-protein synthase in PHYMS anthers agrees with the cross-section results of trinuclear anthers, where, along with clear cellular structures, we observed an obvious endothecium and a thick epidermis.

β-1,3-glucanases, which are known to be expressed just prior to microspore release, are involved in the degradation of callose surrounding the microspore tetrad. In this way, β-1,3-glucanases contribute to the release of microspores as pollen grains [43]. In Petunia, the timing of callose activity is crucial for normal microspore development, and any error in timing can result in male sterility [44, 45]. In our study we identified spot 15, which was downregulated in PHYMS anthers at both the mononuclear and trinuclear stage of development, as endo-β-1,3-glucanase. This finding was consistent with the lower β-1,3-glucanase activity found at each of these stages. In contrast, even though we did not find a significant difference in expression of this protein at the tetrad stage, its activity was still significantly lower in the PHYMS anthers at this stage (Additional file 10: Figure S6a). These results agree with another study which showed that in the CMS system in *Brassica napus*, the activity of β-1,3-glucanase was also higher in normal anthers [26]. Therefore, β-1,3-glucanase likely plays an important role in the process of male sterility in wheat.

Vacuolar invertase plays an integral role in sugar metabolism in higher plants. A decline in vacuolar invertase activity is associated with an accumulation of sucrose, alterations in the profile of other sugars, and a spatial redistribution of starch within anthers [46]. Here, we found vacuolar invertase activity and *Ivr5* expression levels to be significantly lower in PHYMS anthers compared with MF-1376 anthers. This holds true in the mononuclear, and especially in the trinuclear stage of development, and is supported by the results of the 2-D PAGE (Additional file 3: Figure S2, VI and Additional file 10: Figure S6b and c). These results suggest that an abnormal sucrose metabolism involving vacuolar invertase could be correlated with the abortive behavior of PHYMS anthers. This is also supported by the fact that sterile pollen has no accumulation of starch in PHYMS anthers at the trinuclear stage. Our findings also support the increasingly popular idea that invertase-mediated sucrose cleavage in the apoplast of plants is important for supplying carbohydrates to normal developing anthers. Other studies have shown that vacuolar invertase is implicated in pollen abortion in response to drought stress in wheat [47]. Another factor which could contribute to male sterility in PHYMS anthers is a reduced availability of carbohydrates. In our 2-D PAGE experiment, some of the PHYMS spots which had a lower intensity relative to their corresponding MF-1376 spots, were identified as proteins involved in photosynthesis. For example, spot 14, which we identified as chloroplastic RuBisCO large subunit-binding protein subunit alpha (Additional file 3: Figure S2, RLSPSA) had a lower concentration in PHYMS anthers at both the mononuclear and trinuclear stage. RuBisCO large subunit-binding protein subunit alpha is a 65 kDa chaperonin subunit that forms part of larger complex (probably six alpha and six beta subunits) involved in the assembly of RuBisCO in the chloroplast of higher plants [27]. RuBisCO itself, is a key enzyme in the process of photosynthesis. A downregulation of RuBisCO large subunit-binding protein subunit alpha is a clear indication that carbon fixation would be disturbed and the rate of photosynthesis decreased in PHYMS anthers. Due to the lack of these photosynthetic proteins in PHYMS anthers, it is possible that a reduction in the rate of photosynthesis, and therefore a limited availability of carbohydrates, might lead to pollen abortion. Interestingly, RuBisCO large subunit-binding protein subunit was shown to be directly related to the occurrence of male sterility in chili pepper [48].

The ABC transporter family is a large protein family, including complete and half transporter proteins, which have a variety of functions relevant for the transportation of hormones, lipids, metal ions, secondary metabolites, and exogenous substances in plants. As such, it is an extremely important plant membrane transport protein family [49]. We found ABC transporter C family member 5 to be upregulated at the tetrad and mononuclear stage in PHYMS anthers, suggesting that ABC transporters play a role in the process of male sterility induced by CHA. As we identified a transcriptional repressor (spot 11) and a reversibly glycosylated polypeptide (spot 12) in this experiment, it is likely that both transcription regulation and protein modification are also involved in CHA-induced male sterility. In addition, the ubiquitin proteasome pathway (UPP) is known to play an important role in sexual reproduction in higher plants. In this respect, our PPI results combined with previous findings [19] indicate that the ubiquitin proteasome pathway and wheat male sterility are closely related (Figs. 4 and 5).

Based on our proteomics, bioinformatics, and molecular biology results, we propose that the plant is affected either directly or indirectly by the CHA, thereby causing a decline in the rate of photosynthetic,glutathione dispelling heterotoxin requires energy expenditure, a reduction in the starch content due to inactive carbon fixation, and a decrease in vacuole invertase activity. This may in turn lead to a block in sugar metabolic pathways, and consequently a serious shortage of sugar – a compound which is needed for the proper development of microspores and pollen grains. Furthermore, a substantial accumulation of ROS was caused by tapetum cell apoptosis in advance, and the proteasome pathway becomes active and degrades the apoptotic proteins. Thus, transportation pathway of tapetum toward microspores was impeded. Taken together, these mechanisms could lead to carbohydrate starved anthers or pollen, and ultimately male sterility. Certainly, the DEPs observed in this study need to be further characterized in order to determine if they directly affect sterility. We hope that future experiments aimed at specifically downregulating some of these DEPs using CRISPR-CAS9, could help elucidate which proteins are responsible for SQ-1-mediated male sterility.

## Conclusions

Here, we applied a proteomics approach to identify key proteins involved in CHA-induced male sterile in wheat anthers. Our results show that abnormal pollen development in male-sterile anthers is associated with DEPs during the tetrad, mononuclear and/or trinuclear stage. These proteins include oxidative-redox stress proteins (e.g., putative glutathione S-transferase GSTF1), carbon metabolism and cell wall related proteins (e.g., cytochrome c oxidase and vacuolar invertase), proteins involved in photosynthesis (e.g., RuBisCO large subunit-binding protein subunit alpha), and proteins from the ubiquitin proteasome pathway. Importantly, we find that a substantial accumulation of ROS, along with abnormal activities of antioxidant enzymes and ROS-scavenging proteins, leads to chronic oxidative stress and the abortion of microspores in PHYMS plants. We also highlight the importance of the tapetum for normal functioning of the callose (β-1, 3-glucanases) and for pollen development of sugar metabolism (vacuolar invertase). In combination with the phenotypic and tissue section analyses, the proteins we identified in 2D–PAGE indicate that the mechanisms regulating pollen development in PHYMS plants is a complex network. In summary, we provide important clues for understanding the mechanisms of CHA-induced male sterility, and provide insight for the practical application of hybrid breeding in wheat.

## Additional files

Additional file 1: Figure S1. 2-DE patterns of proteins extracted from MF-1376 and PHYMS anthers. (DOCX 1751 kb)

Additional file 2: Table S1. Identification of differentially expressed proteins between MF-1376 and PHYMS anthers. (DOCX 42 kb)

Additional file 3: Figure S2. Analysis of several identified proteins. The readout of the DeCyder Biological Variation Analysis (BVA) module is shown for several proteins. (DOCX 332 kb)

Additional file 4: Figure S3. Hierarchical clustering of identified proteins of all 13 categories and dynamic expression profile for the DEPs. (DOCX 951 kb)

Additional file 5: Table S2a and b. Protein interaction network analysis by searching the STRING 10.0 according to *Brachypodium distachyon* homologous proteins and TAIR homologous proteins, respectively. (DOCX 42 kb)

Additional file 6: Figure S4. Protein interaction network analysis by searching the STRING 10.0 (TAIR homologous proteins). (DOCX 1653 kb)

Additional file 7: Figure S5. Cellular component, molecular function and biological process networks generated by BiNGO. (DOCX 417 kb)

Additional file 8: Table S3. BINGO analysis of differentially expressed proteins. (DOCX 20 kb)

Additional file 9: Dataset S1. The relevant files of ClueGO results. (XLS 607 kb)

Additional file 10: Figure S6. Results of Sugar metabolism related enzyme activity and qRT-PCR. (DOCX 155 kb)

## Abbreviations

2-DE: Two-dimensional electrophoresis
AOX: Alternative oxidase
CAD: Cinnamyl alcohol dehydrogenase
CAT: Catalase
CHA: Chemical hybridizing agents
CMS: Cytoplasmic male sterility
COX: Cytochrome c oxidase
DEPs: Differentially expressed proteins
GMS: Genic male sterility
KI-I_2_: Iodine-potassium iodide solution
MDA: Malondialdehyde
POD: Peroxidase
PPI: Protein–protein interaction
ROS: Reactive oxygen species
SDS-PAGE: Sodium dodecyl sulfate polyacrylamide gel electrophoresis;
SEM: Scanning electron microscopy
SOD: Superoxide dismutase
