# Supplementary material for: Chemical hybridizing agent SQ-1-induced male sterility in Triticum aestivum L.: a comparative analysis of the anther proteome
Source: BMC Plant Biol. 2018 Jan 5;18:7. doi: 10.1186/s12870-017-1225-x (PMC5755283; doi:10.1186/s12870-017-1225-x)
Supplement: Supplementary file 1 — 2-DE patterns of proteins extracted from MF-1376 and PHYMS anthers. (DOCX 1751 kb) [file 12870_2017_1225_MOESM1_ESM.docx]

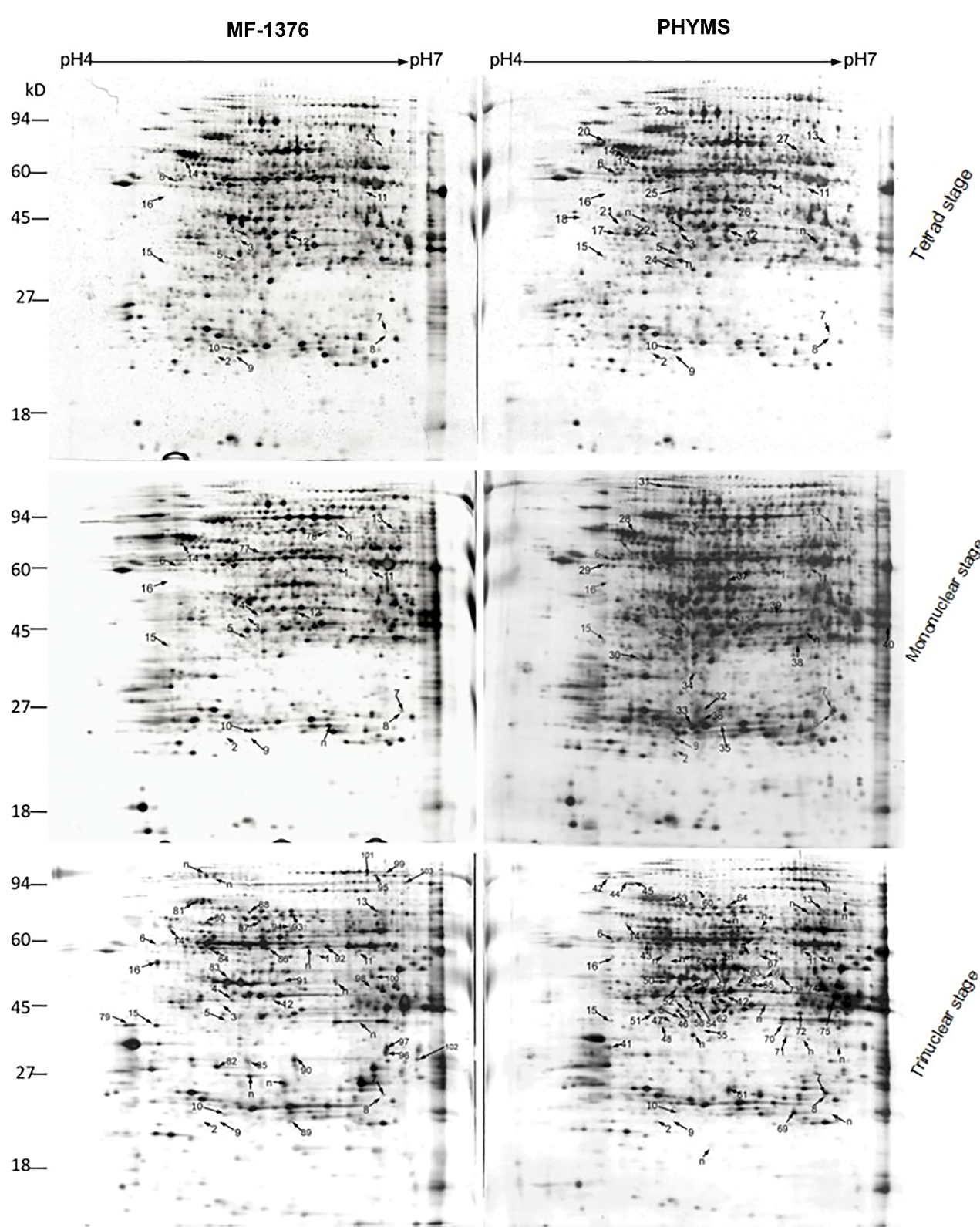


**Figure S1. 2-DE patterns of proteins extracted from MF-1376 and PHYMS anthers.** A protein sample of 300 μg was loaded on each IPG strip (pH 4–7) and protein spots were visualized using silver staining. The experiment was repeated three times, and 16 differentially expressed proteins which common up or down-regulated at tetrad, mononuclear or trinuclear stage showing significant volume changes are labeled on the 2D gel.
