# Supplementary material for: Chemical hybridizing agent SQ-1-induced male sterility in Triticum aestivum L.: a comparative analysis of the anther proteome
Source: BMC Plant Biol. 2018 Jan 5;18:7. doi: 10.1186/s12870-017-1225-x (PMC5755283; doi:10.1186/s12870-017-1225-x)
Supplement: Supplementary file 2 — Identification of differentially expressed proteins between MF-1376 and PHYMS anthers. (DOCX 42 kb) [file 12870_2017_1225_MOESM2_ESM.docx]

**Table S1. Identification of differentially expressed proteins between MF-1376 and PHYMS anthers.**

| ^a^Spot  no. | Protein name | Species | ^b^Accession  no. | ^c^Protein  score | Protein  score  C. I.% | ^d^PC | ^e^TMW/TpI  (kDa) | ^f^EpI/EMW (kDa) | Total Ion  Score/  Total Ion  C. I. % | ^g^Average ratio | | |
| --- | --- | --- | --- | --- | --- | --- | --- | --- | --- | --- | --- | --- |
|  |  |  |  |  |  |  |  |  |  | Tetrad  stage | Mononuclear  stage | Trinuclear  stage |
| Carbohydrate metabolism | | | | | | | | | | | | |
| 16 | vacuolar invertase1 | Triticum monococcum | gi\|46358940 | 374 | 100 | 14 | 71.745/ 4.77 | 55.41/4.85 | 295/100 | 0.41 | 0.75 | 0.07 |
| 19 | Glucose-6-phosphate isomerase | Aegilops tauschii | gi\|475561248 | 293 | 100 | 5 | 67.675/  5.57 | 64.39/5.02 | 224/100 | 1.91 | 1.1 | 1779.42 |
| 24 | PREDICTED: NADH dehydrogenase [ubiquinone] 1beta subcomplex subunit 7-like isoform 1 | Cucumis sativus | gi\|449445262 | 66 | 53 | 7 | 12.09/7.63 | 35.15/5.3 | 22/73 | 2.17 | 1.29 | 508.45 |
| 37 | Dihydrolipoyllysine-residue succinyltransferase component of 2-oxoglutarate dehydrogenase complex 2, mitochondrial | Arabidopsis thaliana | gi\|308197130 | 93 | 99.974 | 6 | 50.028/  9.08 | 54.99/  5.71 | 74/  99.993 | 0.92 | 2.58 | 1.33 |
| 40 | glyceraldehyde-3-phosphate dehydrogenase | Triticum aestivum | gi\|148508784 | 89 | 99.994 | 9 | 36.853/  7.08 | 41.14/  6.99 | 41/  98.932 | 0.67 | 18.44 | 1.53 |
| 43 | UTP--glucose-1-phosphate uridylyltransferase | Triticum urartu | gi\|473993048 | 93 | 99.998 | 9 | 51.083/  5.76 | 61.7/  5.1 | 46/  99.806 | 1.22 | 0.77 | 2.32 |
| 49 | Succinyl-CoA ligase [ADP-forming] subunit beta,mitochondria | Triticum urartu | gi\|474416303 | 174 | 100 | 12 | 45.498/  6.5 | 45/  5.4 | 112/100 | 1.02 | 1.07 | 1.61 |
| 64 | phosphoglucomutase | Triticum aestivum | gi\|18076790 | 145 | 100 | 16 | 62.978/  5.66 | 77.31/  5.74 | 45/  99.767 | 0.43 | 1.03 | 1.7 |
| 66 | Isocitrate dehydrogenase [NADP], chloroplastic | Triticum urartu | gi\|473933356 | 64 | 98.357 | 1 | 47.717/  5.99 | 49.15/  6.04 | 52/  99.66 | 0.78 | 1.16 | 2.23 |
| 73 | Isocitrate dehydrogenase [NADP], chloroplastic | Triticum urartu | gi\|473933356 | 121 | 100 | 15 | 47.717/  5.99 | 48.82/  6.19 | 24/63.251 | 0.73 | 1.13 | 1.59 |
| 74 | alcohol dehydrogenase ADH1A subsp. dicoccon | Triticum turgidum | gi\|119388731 | 204 | 100 | 6 | 41.729/  6.15 | 46.75/  6.5 | 140/100 | 1.19 | 1.02 | 4.77 |
| 75 | glyceraldehyde-3-phosphate dehydrogenase | Triticum aestivum | gi\|253783729 | 386 | 100 | 9 | 36.619/  6.67 | 43.89/  6.61 | 328/100 | 0.65 | 1.97 | 2.76 |
| 79 | Glucan endo-1,3-beta-glucosidase GII | Triticum urartu | gi\|473895095 | 54 | 98.697 | 3 | 34.078/  4.72 | 37.32/  4.63 | 43/  99.791 | 209.07 | 0.34 | 0.17 |
| 84 | UTP--glucose-1-phosphate uridylyltransferase | Triticum urartu | gi\|473993048 | 355 | 100 | 17 | 51.083/  5.76 | 61.13/  5.24 | 228/100 | 1.19 | 1.64 | 0.37 |
| 88 | phosphoglucomutase | Triticum aestivum | gi\|18076790 | 43 | 100 | 10 | 62.977/  5.66 | 76.65/  5.65 | 0/0 | 0.82 | 0.8 | 0.18 |
| 92 | UTP--glucose-1-phosphate uridylyltransferase | Aegilops tauschii | gi\|475574945 | 177 | 100 | 14 | 47.521/  6.63 | 61.85/  6.13 | 56/  99.532 | 0.54 | 2.04 | 0.3 |
| 95 | 4-alpha-glucanotransferase DPE2 | Triticum urartu | gi\|474139599 | 67 | 100 | 17 | 101.321/  6.21 | 96.81/  6.48 | 83/100 | 393.73 | 0.34 | 0.27 |
| 99 | 4-alpha-glucanotransferase DPE2 | Triticum urartu | gi\|474139599 | 315 | 100 | 24 | 101.321/  6.21 | 96.69/  6.55 | 158/100 | 566.99 | 0.34 | 0.33 |
| Mitochondrial electron transport/ATP synthesis | | | | | | | | | | | | |
| 1 | Putative cytochrome c oxidase subunit II PS17  (Fragments) | Pinus strobus | PS17_PINST | 79 | 99.35 | 2 | 1.707/9.63 | 58.68/6.06 | 56/99.449 | 2.19 | 3.17 | 4.74 |
| 10 | alternative oxidase | Triticum aestivum | gi\|19912725 | 64 | 98.114 | 10 | 36.784/8.67 | 24.07/5.30 | 16/0 | 1.14 | 580.94 | 1045.82 |
| 26 | Putative cytochrome c oxidase subunit II PS17(Fragments) OS=Pinus strobus PE=1 SV=1 | Pinus strobus | PS17_PINST | 92 | 99.969 | 2 | 1.7/9.63 | 50.62/5.75 | 43/76.757 | 1.93 | 1.83 | 2.18 |
| 55 | Putative cytochrome c oxidase subunit II PS17(Fragments) | Pinus strobus PE=1 SV=1 | PS17_PINST | 82 | 99.659 | 2 | 1.707/  9.63 | 36.57/5.51 | 0/0 | 1 | 1.01 | 4.58 |
| 93 | NADP-dependent malic enzyme | Triticum aestivum | gi\|158701881 | 108 | 100 | 13 | 63.391/  5.56 | 75.28/  5.87 | 33/  94.018 | 0.54 | 0.73 | 0.41 |
| Metabolism of others secondary metabolites | | | | | | | | | | | | |
| 2 | ABC transporter C family member 5 | Triticum urartu | gi\|474343757 | 56 | 89.392 | 16 | 139.3/8.17 | 23.48/5.16 | 23 /30.634 | 9.47 | 18.94 | 2.52 |
| 3 | Putative cinnamyl alcohol dehydrogenase 8D | Triticum urartu | gi\|473822195 | 103 | 100 | 4 | 41.449/5.71 | 43.10/5.33 | 88/100 | 2.79 | 4.56 | 2.5 |
| 22 | Diphosphomevalonate decarboxylase | Triticum urartu | gi\|474368068 | 52 | 73.96 | 9 | 32.728/  7.63 | 44.97/  5.11 | 0/0 | 2.43 | 1.35 | 1.08 |
| 30 | Stilbene synthase 4 | Triticum urartu | gi\|474152910 | 112 | 100 | 7 | 42.106/  5.34 | 34.02/  5.04 | 78/100 | 0.71 | 2.15 | 464.59 |
| 52 | ABC transporter C family member 10 | Triticum urartu | gi\|473846061 | 88 | 99.906 | 7 | 173.990/  8.07 | 45.02/  5.28 | 45/  85.854 | 2.05 | 2.02 | 2.62 |
| Cytoskeleton and cell structrue | | | | | | | | | | | | |
| 4 | Alpha-1,4-glucan-protein synthase [UDP-forming] 1 | Triticum urartu | gi\|474042704 | 94 | 99.998 | 6 | 63.955/5.55 | 43.15/5.38 | 76 /100 | 1.52 | 1.57 | 2.04 |
| 6 | beta-tubulin 3 | Triticum aestivum | gi\|4098323 | 272 | 100 | 10 | 50.554/4.67 | 62.08/4.85 | 214/100 | 1.32 | 2.35 | 5.15 |
| 12 | reversibly glycosylated polypeptide | Triticum aestivum | gi\|4158232 | 310 | 100 | 14 | 41.985/5.82 | 43.41/5.72 | 201/100 | 0.34 | 0.61 | 1.94 |
| 15 | endo-beta-1,3-glucanase | Triticum aestivum | gi\|109150356 | 225 | 100 | 11 | 35.039/4.86 | 37.28/4.84 | 147/100 | 1.72 | 0.69 | 0.14 |
| 17 | Putative cellulose synthase-like protein D5 | Triticum urartu | gi\|474072613 | 55 | 86.645 | 10 | 86.134/  8.71 | 42.66/  4.9 | 33/24.865 | 1.52 | 0.75 | 2.07 |
| 35 | cellulose synthase-like protein E1 | Arabidopsis thaliana | gi\|22330270 | 66 | 77.701 | 13 | 83.548/  6.19 | 24.41/  5.73 | 0/0 | 0 | 3.21 | 0.57 |
| 83 | Actin | Triticum aestivum | gi\|58533119 | 164 | 100 | 8 | 41.929/  5.23 | 48.78/  5.35 | 121/100 | 0.22 | 0.86 | 0.27 |
| 89 | PREDICTED: wall-associated receptor kinase 5-like | Solanum lycopersicum | gi\|460408572 | 51 | 95.038 | 6 | 23.876/  5.8 | 23.17/  5.85 | 0/0 | 1.14 | 0.34 | 0.1 |
| 90 | histone H1 subtype 7 | Pisum sativum subsp. elatius | gi\|528066018 | 129 | 100 | 7 | 19.561/  10.8 | 30.61/  5.89 | 108/100 | 1.14 | 0.38 | 0.09 |
| 94 | waxy D1, partial | Triticum spelta | gi\|310619520 | 71 | 99.68 | 14 | 64.035/  8.62 | 70.24/  5.85 | 0/0 | 1.14 | 0.34 | 0.25 |
| 101 | Vacuolar-sorting receptor 4 | Triticum urartu | gi\|474446079 | 59 | 97.739 | 2 | 30.734/  4.89 | 96.84/  6.42 | 33/58.702 | 1.14 | 0.34 | 0 |
| Oxidative stress/resistance | | | | | | | | | | | | |
| 7 | putative glutathione S-transferase GSTF1 | Triticum urartu | gi\|473881465 | 178 | 100 | 11 | 25.084/5.99 | 25.17/6.54 | 90/100 | 2.46 | 2.26 | 5.47 |
| 8 | superoxide dismutase | Triticum aestivum | gi\|226897531 | 118 | 100 | 4 | 23.184/5.75 | 25.36/6.53 | 54/99.394 | 2.23 | 2.62 | 10.08 |
| 9 | putative In2.1 protein | Triticum aestivum | gi\|3393062 | 60 | 95.151 | 4 | 27.249/5.38 | 23.35/5.32 | 38 /98.008 | 425.91 | 543.53 | 838.23 |
| 13 | 66 kDa stress protein | Triticum urartu | gi\|473980005 | 154 | 100 | 11 | 59.619/6.08 | 77.28/6.50 | 94/100 | 0.46 | 0.43 | 0.1 |
| 36 | glutathione transferase F3 | Triticum aestivum | gi\|23504741 | 111 | 100 | 5 | 24.642/  5.33 | 24.91/  5.52 | 79/100 | 0 | 5.4 | 1336.4 |
| 44 | heat shock protein 90 | Triticum dicoccoides | gi\|294717871 | 58 | 92.661 | 11 | 80.695/  4.96 | 88.88/  4.92 | 17/0 | 183.79 | 0.34 | 5.62 |
| 45 | Heat shock protein 81-3 | Triticum urartu | gi\|474049119 | 195 | 100 | 15 | 80.668/  4.96 | 89.67/  4.99 | 112/100 | 1.14 | 0.95 | 5.7 |
| 53 | HSP70 | Triticum aestivum | gi\|2827002 | 214 | 100 | 13 | 71.358/  5.15 | 81.24/  5.23 | 158/100 | 3.85 | 0.56 | 2.54 |
| 56 | Bifunctional polymyxin resistance protein ArnA | Triticum urartu | gi\|474224464 | 79 | 99.943 | 8 | 43.507/  7.53 | 51.6/  5.48 | 40/  99.158 | 1.63 | 0.96 | 2.28 |
| 59 | TMV resistance protein N, putative | Ricinus communis | gi\|223543328 | 75 | 99.625 | 12 | 136.187/  5.55 | 55.37/  5.55 | 45/  87.157 | 2.76 | 1.4 | 4.27 |
| 61 | Serine/arginine-rich splicing factor 12 | Triticum urartu | gi\|474094791 | 36 | 99.301 | 16 | 104.384/  9.58 | 25.85/  5.76 | 47.03/37 | 1061.32 | 1.53 | 5.86 |
| 63 | S-adenosylmethionine synthase 1 | Triticum urartu | gi\|474105890 | 201 | 100 | 9 | 43.217/  5.61 | 52.08/  5.84 | 146/  100 | 1.56 | 1.15 | 2.67 |
| 69 | glutathione S-transferase | Triticum aestivum | gi\|5923877 | 63 | 99.732 | 4 | 23.609/  5.79 | 23.75/  6.28 | 40/99.358 | 1.06 | 0.96 | 1.69 |
| 81 | Heat shock cognate 70 kDa protein 1 | Triticum urartu | gi\|474012573 | 143 | 100 | 17 | 71478/  5.07 | 81.27/  5.11 | 55/99.969 | 1.33 | 0 | 0.14 |
| 91 | PREDICTED: cytochrome P450 82C4-like | Solanum lycopersicum | gi\|460382941 | 76 | 97.409 | 6 | 19.275/  5.21 | 48.07/  5.8 | 43/57 | 0.76 | 0.74 | 0.19 |
| 98 | Peroxidase 65 | Triticum urartu | gi\|473843112 | 88 | 99.993 | 12 | 39.618/  6.24 | 46.29/  6.45 | 0/0 | 1.14 | 0.34 | 0.32 |
| 100 | Peroxidase 65 | Triticum urartu | gi\|473843112 | 213 | 100 | 15 | 39.618/  6.24 | 50.36/  6.46 | 89/100 | 1.52 | 0.32 | 0 |
| 102 | DNAJ heat shock N-terminal domain-containing protein | Arabidopsis thaliana | gi\|30688675 | 57 | 0 | 13 | 123.961/  8.4 | 30.71/  6.79 | 0/0 | 1.14 | 0.34 | 0 |
| Photosynthesis | | | | | | | | | | | | |
| 14 | RuBisCO large subunit-binding protein subunit alpha,  chloroplastic | Triticum urartu | gi\|474113969 | 686 | 100 | 24 | 65.625/5.17 | 70.03/4.94 | 486/100 | 1.79 | 0.64 | 0.24 |
| 21 | Magnesium-chelatase subunit chlI, chloroplastic | Triticum urartu | gi\|474036467 | 71 | 99.649 | 8 | 45.5/  5.4 | 44.09/  4.92 | 35/  97.782 | 1.89 | 0.92 | 16.9 |
| 29 | ribulose-1,5-bisphosphate carboxylase/oxygenase large subunit (chloroplast) | Triticum monococcum | gi\|525778513 | 101 | 100 | 15 | 53.344/  6.04 | 62.08/  4.85 | 4/0 | 1.32 | 2.35 | 5.15 |
| 46 | chloroplast fructose-bisphosphate aldolase | Triticum aestivum | gi\|223018643 | 107 | 10 | 4 | 42.216/  5.94 | 39.75/  5.32 | 93/100 | 0.69 | 0.82 | 1.83 |
| 60 | Transketolase, chloroplastic | Aegilops tauschii | gi\|475481099 | 169 | 100 | 2 | 69.455/  5.36 | 85.53/  5.49 | 153/100 | 0.88 | 1.01 | 2.35 |
| 62 | ribulose-1,5-bisphosphate carboxylase/oxygenase larges ubunit (chloroplast) | Triticum monococcum | gi\|525778513 | 124 | 100 | 10 | 53.343/  6.04 | 40.08/  5.73 | 72/100 | 1.56 | 1.16 | 2.58 |
| 72 | ribulose-1,5-bisphosphate carboxylase/oxygenase large subunit (chloroplast) | Triticum monococcum | gi\|525778513 | 68 | 99.346 | 11 | 53.343/  6.04 | 39.65/  6.33 |  | 1.2 | 0.89 | 2.83 |
| Proteins metabolism | | | | | | | | | | | | |
| 23 | Subtilisin-like protease SDD1 | Triticum urartu | gi\|474286973 | 150 | 100 | 11 | 81.549/  5.95 | 89.1/5.31 | 105/100 | 2.17 | 1.39 | 1.92 |
| 25 | Eukaryotic initiation factor 4A-1 | Aegilops tauschii | gi\|475619612 | 117 | 100 | 11 | 47.158/5.38 | 56.1/5.32 | 42/84.675 | 1.6 | 1.11 | 885.5 |
| 28 | protein disulfide isomerase | Triticum aestivum | gi\|335906213 | 357 | 100 | 23 | 56.497/  5.06 | 73.5/4.99 | 164/100 | 1.06 | 3.68 | 1.05 |
| 32 | Cyanelle 30S ribosomal protein S16 OS=Cyanophora paradoxa GN=rps16 PE=3 SV=1 | Cyanophora paradoxa | RR16_CYAPA | 58 | 20.02 | 3 | 8.886/  11 | 26.27/  5.5 | 43/92.003 | 0.97 | 4.11 | 1250.07 |
| 33 | Peptide chain release factor 1 | Desulfovibrio salexigens | RF1_DESAD | 78 | 99.182 | 13 | 40.324/  5.1 | 25.11/  5.35 | 0/0 | 2.23 | 3.74 | 1.08 |
| 50 | retrotransposon protein, putative, Ty1-copia subclass | Oryza sativa Japonica Group | gi\|77554581 | 80 | 99.957 | 17 | 96.256/  8.8 | 46.94/  5.25 |  | 1.82 | 0.55 | 3.24 |
| 65 | Elongation factor Tu, mitochondrial | Triticum urartu | gi\|474103525 | 103 | 100 | 9 | 43.723/  5.67 | 46.14/  5.97 | 52/  99.965 | 1.18 | 1.13 | 1.65 |
| 67 | 26S protease regulatory subunit 6B-like protein | Triticum urartu | gi\|473825073 | 70 | 99.547 | 11 | 41.599/  5.91 | 56.83/  6.02 |  | 1.25 | 1.08 | 2.31 |
| 68 | Elongation factor Tu, mitochondrial | Triticum urartu | gi\|474103525 | 72 | 99.708 | 1 | 43.722/  5.67 | 46.04/  5.84 | 64/99.992 | 0.98 | 1.37 | 1.86 |
| 71 | 26S proteasome non-ATPase regulatory subunit 14 | Triticum urartu | gi\|474213923 | 50 | 99.778 | 2 | 32.331/  5.61 | 34.31/  6.21 | 31/70.806 | 0.72 | 0.92 | 4.33 |
| 76 | F-box associated ubiquitination effector family protein | Arabidopsis thaliana | gi\|22330325 | 57 | 99.865 | 4 | 16.807/  6.59 | 23.56/  4.24 | 23/56 | 0.97 | 0.16 | 1.08 |
| 86 | Mitochondrial-processing peptidase subunit alpha | Triticum urartu | gi\|474069724 | 97 | 100 | 10 | 41.700/  5.49 | 60.97/  5.66 | 19/0 | 0.57 | 2.51 | 0.28 |
| 97 | 26S proteasome ATPase subunit | Triticum aestivum | gi\|32400818 | 77 | 95.982 | 4 | 32.087/  6.43 | 32.81/  6.57 | 23/  69.235 | 0.51 | 1.43 | 0.11 |
| Lipid metabolism | | | | | | | | | | | | |
| 27 | Fatty acyl-CoA reductase 2 | Aegilops tauschii | gi\|475620279 | 53 | 100 | 11 | 65.487/5.85 | 72.72/6.28 | 39/88.35 | 1.93 | 1.01 | 0.51 |
| 96 | 1-acyl-sn-glycerol-3-phosphate acyltransferase 1 | Arabidopsis thaliana | gi\|332660383 | 54 | 99.453 | 9 | 39.768/  9.87 | 31.89/  6.56 | 57/  88.374 | 1.07 | 0.66 | 0.12 |
| Signal | | | | | | | | | | | | |
| 47 | P-glycoprotein 18 | Arabidopsis lyrata subsp. lyrata | gi\|297322942 | 71 | 91.904 | 16 | 129.407/  8.68 | 37.16/5.26 | 0/0 | 1.2 | 0.84 | 6.59 |
| 77 | Ca2+/calmodulin-dependent protein kinase, EF-Hand protein superfamily (ISS) | Ostreococcus tauri | gi\|308808452 | 56 | 95.701 | 13 | 66.882/  6.76 | 66.1/  5.43 | 39/71 | 1.14 | 0.4 | 707.31 |
| 78 | calcium-dependent protein kinase, putative | Ricinus communis | gi\|223526474 | 53 | 96.48 | 12 | 59.860/  5.82 | 75.71/  5.99 | 17/33 | 1.94 | 0.54 | 1.08 |
| 87 | PREDICTED: calmodulin-like protein 7-like | Solanum lycopersicum | gi\|460393692 | 53 | 95.032 | 7 | 17.051/  4.3 | 72.74/  5.6 | 0/0 | 0 | 1.12 | 0.34 |
| 34 | putative ADP-ribosylation factor GTPase-activating protein AGD11 | Triticum urartu | gi\|474305481 | 62 | 97.396 | 2 | 21.213/  5.94 | 31.07/  5.45 | 30/  58.845 | 1.14 | 2.78 | 1/08 |
| 31 | Cell division cycle protein 48-like protein | Triticum urartu | gi\|473821746 | 105 | 100 | 17 | 91.507/  5.13 | 98.25/  5.21 | 28/  88.173 | 2.27 | 1.58 | 546.58 |
| Transcription | | | | | | | | | | | | |
| 11 | Transcriptional repressor NrdR | Dechloromonas  aromatica (strain RCB) | NRDR_DECAR | 73 | 97.352 | 9 | 17.769/9.39 | 58.09/6.30 | 47/95.857 | 3.57 | 396.92 | 1.08 |
| 48 | Transcription-associated protein 1 | Triticum urartu | gi\|473940347 | 52 | 72.098 | 29 | 429.655/  6.66 | 36.77/  5.18 |  | 1.62 | 0 | 3.2 |
| 70 | putative histone acetyltransferase HAC-like 1 | Triticum urartu | gi\|474215427 | 55 | 85.69 | 10 | 32.932/  9.26 | 37.31/  6.17 |  | 1.21 | 1.35 | 2.52 |
| 80 | RNA-binding protein Nova-1 | Aegilops tauschii | gi\|475532979 | 61 | 95.021 | 4 | 9.608/  5.53 | 71.89/  5.21 | 26/88.3 | 0.6 | 0.75 | 0.36 |
| Amino acid metabolism | | | | | | | | | | | | |
| 54 | Arginase | Triticum urartu | gi\|474404660 | 245 | 100 | 6 | 37.139/  5.62 | 41.24/  5.51 | 199/100 | 0 | 0.34 | 2.48 |
| 57 | Fumarylacetoacetase | Triticum urartu | gi\|473796898 | 183 | 100 | 7 | 47.338/  5.48 | 52.07/  5.61 | 136/100 | 1.55 | 0.59 | 3.17 |
| Others | | | | | | | | | | | | |
| 39 | expressed protein | Oryza sativa Japonica Group | gi\|77552263 | 69 | 88.297 | 12 | 35.828/  7.67 | 43.45/  6.09 | 0/0 | 1.48 | 3.4 | 11.02 |
| 58 | Glutelin type-A 2 | Aegilops tauschii | gi\|475560198 | 225 | 100 | 3 | 38.139/  5.6 | 39.68/  5.46 | 202/100 | 1.33 | 1.05 | 2.24 |
| 82 | Pollen allergen KBG 41 | Triticum urartu | gi\|473728248 | 73 | 96.302 | 2 | 30.047/  6.64 | 29.42/  5.28 | 29/94.107 | 1.14 | 0.34 | 0.26 |
| 85 | group 5/9 grass pollen allergen R8-5, partial | Secale cereale x Triticum durum | gi\|365769201 | 86 | 99.782 | 5 | 24.911/  5.61 | 30.38/  5.51 | 58/  99.757 | 0.76 | 0.97 | 0.14 |
| Unknown | | | | | | | | | | | | |
| 5 | Unnamed protein product | Triticum aestivum | gi\|259662485 | 280 | 100 | 10 | 31.422/5.06 | 36.67/5.30 | 210/100 | 3.62 | 1.86 | 2.35 |
| 18 | hypothetical protein CARUB_v10009038mg | Capsella rubella | gi\|482576126 | 74 | 96.213 | 15 | 54.346/  9.37 | 46.42/  4.62 | 51/62.361 | 2.8 | 21.22 | 0.59 |
|  |  |  |  |  |  |  |  |  |  |  |  |  |
| 20 | hypothetical protein F775_30457 | Aegilops tauschii | gi\|475493180 | 157 | 100 | 12 | 55.533/  4.74 | 75.1/  4.78 | 87/100 | 2.16 | 4.26 | 473.87 |
| 38 | hypothetical protein F775_29537 | Aegilops tauschii | gi\|475620773 | 79 | 98.856 | 9 | 33.107/  5.96 | 37/  6.26 | 0/0 | 1.35 | 1.72 | 141.87 |
| 41 | hypothetical protein OsI_21172 | Oryza sativa Indica Group | gi\|125553505 | 66 | 72.567 | 11 | 38.942/  9.35 | 31.79/  4.83 | 0/0 | 1.19 | 0.98 | 2.98 |
| 42 | unnamed protein product | Vitis vinifera | gi\|296088147 | 73 | 94.773 | 17 | 100.791/  7.59 | 93.54/  4.76 | 0/0 | 3.33 | 0.86 | 2.71 |
| 51 | hypothetical protein TRIUR3_22532 | Triticum urartu | gi\|473797438 | 89 | 99.994 | 9 | 36.043/  5.13 | 39.23/  5.12 | 32/  92.132 | 1.08 | 0.76 | 3.26 |
| 103 | hypothetical protein TRIUR3_23970 | Triticum urartu | gi\|473813413 | 127 | 100 | 4 | 30.784/  6.17 | 92.33/  6.7 | 89/100 | 1.14 | 0.34 | 0 |

^a^Spot numbers correspond with 2-D gel as shown in supplementary Figure S1.

^b^Accession number in NCBI database or Uniprot.

^c^Protein score was based on combined MS and MS/MS spectra. The proteins that had a statistically significant (p < 0.05) protein score of 50-55 or more were considered successfully identified.

^d^PC: matched peptide count

^e^TpI/TMW (kDa): pI of predicted protein/molecular mass of predicted protein .

^f^EpI/EMW (kDa): pI of protein on the gel/molecular mass of protein on the gel.

^g^Average ratio: average ratio of the protein abundance (PHYMS / MF-1376) on different anther stages.
