# Supplementary material for: Chemical hybridizing agent SQ-1-induced male sterility in Triticum aestivum L.: a comparative analysis of the anther proteome
Source: BMC Plant Biol. 2018 Jan 5;18:7. doi: 10.1186/s12870-017-1225-x (PMC5755283; doi:10.1186/s12870-017-1225-x)
Supplement: Supplementary file 3 — Analysis of several identified proteins. The readout of the DeCyder Biological Variation Analysis (BVA) module is shown for several proteins. (DOCX 332 kb) [file 12870_2017_1225_MOESM3_ESM.docx]

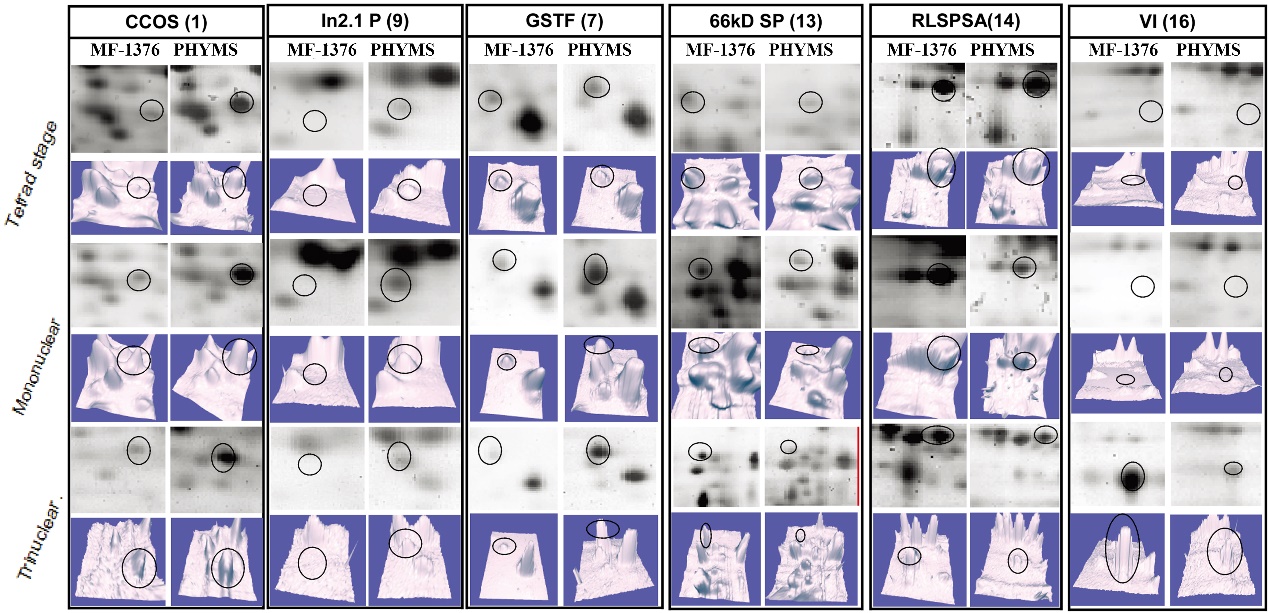


**Figure S2. Analysis of several** **identified proteins.** The readout of the DeCyder Biological Variation Analysis (BVA) module is shown for Putative cytochrome c oxidase subunit II PS17 (Fragments) (CCOS, spot 1), putative glutathione S-transferase GSTF1 (GSTF, spot No.7), putative In2.1 protein (In 2.1 P, spot No. 9), 66 kDa stress protein (66kDa SP, spot No.13), RuBisCO large subunit-binding protein subunit alpha, chloroplastic (RLSPSA, spot No. 14) and vacuolar invertase1 (VI, spot No. 16). Enlarged regions of 2D-PAGE gels for MF-1376 and PHYMS anthers protein, and the corresponding 3D views, are represented.
