# Supplementary material for: Chemical hybridizing agent SQ-1-induced male sterility in Triticum aestivum L.: a comparative analysis of the anther proteome
Source: BMC Plant Biol. 2018 Jan 5;18:7. doi: 10.1186/s12870-017-1225-x (PMC5755283; doi:10.1186/s12870-017-1225-x)
Supplement: Supplementary file 4 — Hierarchical clustering of identified proteins of all 13 categories and dynamic expression profile for the DEPs. (DOCX 951 kb) [file 12870_2017_1225_MOESM4_ESM.docx]

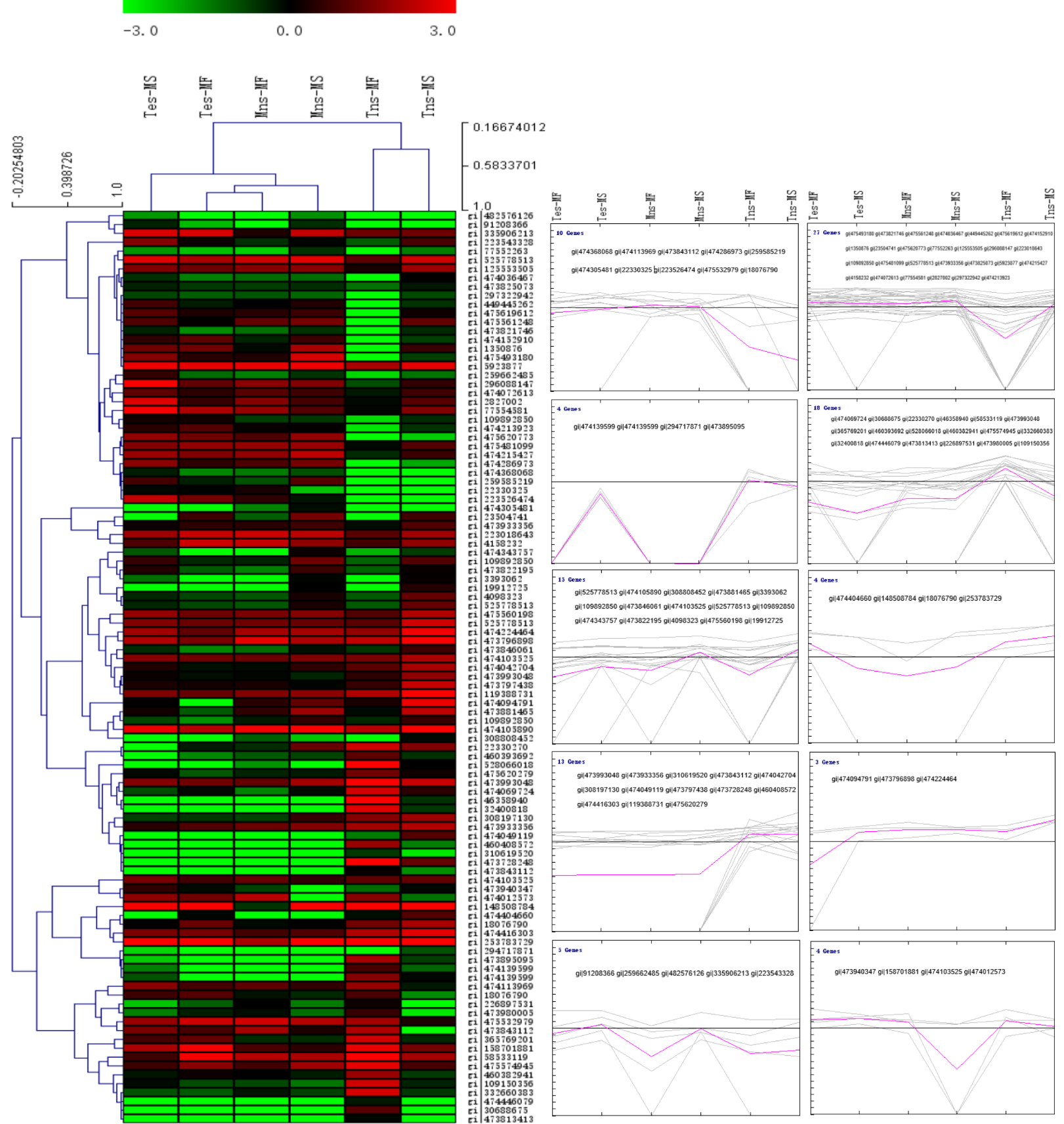


**Figure S3. Hierarchical clustering of identified proteins of all 13 categories and dynamic expression profile for the DEPs.** Tes-MF: the tetrad stage of MF-1376; Tes-MS: the tetrad stage of PHYMS; Mns-MF: the mononuclear stage of MF-1376; Mns-MS: the mononuclear stage of PHYMS; Tns-MF: the trinuclear stage of MF-1376; Tns-MS: the trinuclear stage of PHYMS.
