# Supplementary material for: Chemical hybridizing agent SQ-1-induced male sterility in Triticum aestivum L.: a comparative analysis of the anther proteome
Source: BMC Plant Biol. 2018 Jan 5;18:7. doi: 10.1186/s12870-017-1225-x (PMC5755283; doi:10.1186/s12870-017-1225-x)
Supplement: Supplementary file 5 — Protein interaction network analysis by searching the STRING 10.0 according to Brachypodium distachyon homologous proteins and TAIR homologous proteins, respectively. (DOCX 42 kb) [file 12870_2017_1225_MOESM5_ESM.docx]

**Table S2a. Protein interaction network analysis by searching the STRING 10.0 according to brachypodium distachyon homologous proteins.**

| **Accession number** | **Brachypodium distachyon homologous proteins** | **STRING Name** | **Protein Name** | **Description** |
| --- | --- | --- | --- | --- |
| gi\|473881465\|gb\|EMS47979.1\| | XP_003567701.1 | BRADI2G13110.1 | probable glutathione S-transferase GSTF1-like | probable glutathione S-transferase GSTF1-like (219 aa) |
| gi\|226897531\|gb\|ACO90195.1\| | XP_003558478.1 | BRADI1G69680.1 | superoxide dismutase [Cu-Zn] 2-like | Destroys radicals which are normally produced within the cells and which are toxic to biological systems (By similarity) (164 aa) |
| gi\|109892850\|sp\|P84733.1\|PS17_PINST | XP_003575554.1 | BRADI4G07997.1 | E3 ubiquitin-protein ligase UPL1-like | E3 ubiquitin-protein ligase UPL1-like (3636 aa) |
| gi\|473822195\|gb\|EMS47048.1\| | XP_003578087.1 | BRADI4G29780.1 | probable cinnamyl alcohol dehydrogenase 8C-like | probable cinnamyl alcohol dehydrogenase 8C-like (421 aa) |
| gi\|474042704\|gb\|EMS53325.1\| | XP_003562725.1 | BRADI1G21990.1 | UDP-arabinopyranose mutase 3-like | UDP-arabinopyranose mutase 3-like (363 aa) |
| gi\|4098323\|gb\|AAD10489.1\| | XP_003569962.1 | BRADI3G05010.1 | tubulin beta-1 chain-like | Tubulin is the major constituent of microtubules. It binds two moles of GTP, one at an exchangeable site on the beta chain and one at a non-exchangeable site on the alpha chain (By similarity) (445 aa) |
| gi\|226897531\|gb\|ACO90195.1\| | XP_003558478.1 | BRADI1G69680.1 | superoxide dismutase [Cu-Zn] 2-like | Destroys radicals which are normally produced within the cells and which are toxic to biological systems (By similarity) (164 aa) |
| gi\|3393062\|emb\|CAA76758.1\| | XP_003558194.1 | BRADI1G66030.1 | protein IN2-1 homolog B-like | protein IN2-1 homolog B-like (301 aa) |
| gi\|19912725\|dbj\|BAB88645.1\| | XP_003580438.1 | BRADI5G20540.1 | alternative oxidase 1a, mitochondrial-like | alternative oxidase 1a, mitochondrial-like (333 aa) |
| gi\|91208366\|sp\|Q47IH0.1\|NRDR_DECAR | XP_003558600.1 | BRADI1G71530.1 | LL-diaminopimelate aminotransferase, chloroplastic-like | LL-diaminopimelate aminotransferase, chloroplastic-like (437 aa) |
| gi\|474072613 | XP_003568676.1 | BRADI2G03380.1 | cellulose synthase-like protein D5-like | cellulose synthase-like protein D5-like (997 aa) |
| gi\|482576126\|gb\|EOA40313.1 | XP_003573405.1 | BRADI3G01700.1 | uncharacterized LOC100844385 | uncharacterized LOC100844385 (459 aa) |
| gi\|475561248\|gb\|EMT13737.1\| | XP_003578256.1 | BRADI4G32530.1 | glucose-6-phosphate isomerase-like | glucose-6-phosphate isomerase-like (623 aa) |
| gi\|475493180\|gb\|EMT03692.1\| | XP_003575373.1 | BRADI3G48920.1 | uncharacterized LOC100832360 | uncharacterized LOC100832360 (708 aa) |
| gi\|474036467\|gb\|EMS53132.1\| | XP_003557120.1 | BRADI1G49770.1 | magnesium-chelatase subunit chlI, chloroplastic-like | magnesium-chelatase subunit chlI, chloroplastic-like (417 aa) |
| gi\|474368068\|gb\|EMS63646.1\| | XP_003565147.1 | BRADI2G61850.1 | diphosphomevalonate decarboxylase-like | diphosphomevalonate decarboxylase-like (417 aa) |
| gi\|474286973\|gb\|EMS61151.1\| | XP_003570499.1 | BRADI3G57130.1 | subtilisin-like protease-like | subtilisin-like protease-like (780 aa) |
| gi\|449445262\|ref\|XP_004140392.1\| | XP_003558900.1 | BRADI1G76450.1 | NADH dehydrogenase [ubiquinone] 1 beta  subcomplex subunit 7-like | NADH dehydrogenase [ubiquinone] 1 beta subcomplex subunit 7-like (111 aa) |
| gi\|475619612\|gb\|EMT30822.1\| | XP_003574572.1 | BRADI3G03710.1 | eukaryotic initiation factor 4A-1-like | eukaryotic initiation factor 4A-1-like (414 aa) |
| gi\|475620279\|gb\|EMT31062.1\| | XP_003578640.1 | BRADI4G37860.1 | fatty acyl-CoA reductase 1-like | fatty acyl-CoA reductase 1-like (497 aa) |
| gi\|335906213\|gb\|AEH68230.1\| | XP_003577736.1 | BRADI4G23180.1 | protein disulfide-isomerase-like | protein disulfide-isomerase-like (518 aa) |
| gi\|525778513\|ref\|YP_008239101.1\| | YP_002000495.1 | BrdiC_p033 | ribulose-1,5-bisphosphate carboxylase/oxygenase large subunit | RuBisCO catalyzes two reactions- the carboxylation of D- ribulose 1,5-bisphosphate, the primary event in carbon dioxide fixation, as well as the oxidative fragmentation of the pentose substrate in the photorespiration process. Both reactions occur simultaneously and in competition at the same active site (By similarity) (458 aa) |
| gi\|474152910\|gb\|EMS57002.1\| | XP_003574084.1 | BRADI3G29230.1 | stilbene synthase 2-like | stilbene synthase 2-like (386 aa) |
| gi\|473821746\|gb\|EMS47029.1\| | XP_003558791.1 | BRADI1G74922.1 | cell division control protein 48 homolog E-like | cell division control protein 48 homolog E-like (811 aa) |
| gi\|1350876\|sp\|P48139.1\|RR16_CYAPA | XP_003578361.1 | BRADI4G34170.1 | 30S ribosomal protein S16-like | 30S ribosomal protein S16-like (139 aa) |
| gi\|259585219\|sp\|C6BYQ4.1\|RF1_DESAD | XP_003568985.1 | BRADI2G36930.1 | peptide chain release factor 1-like | peptide chain release factor 1-like (417 aa) |
| gi\|474305481\|gb\|EMS61741.1\| | XP_003563063.1 | BRADI1G26850.1 | probable ADP-ribosylation factor GTPase-activating  protein AGD11-like | probable ADP-ribosylation factor GTPase-activating protein AGD11-like (168 aa) |
| gi\|22330270\|ref\|NP_175981.2\| | XP_003576649.1 | BRADI4G33090.1 | cellulose synthase-like protein E6-like | cellulose synthase-like protein E6-like (725 aa) |
| gi\|23504741\|emb\|CAD29476.1\| | XP_003566561.1 | BRADI2G35950.1 | glutathione S-transferase 4-like | glutathione S-transferase 4-like (226 aa) |
| gi\|308197130\|sp\|Q8H107.2\|ODO2B_ARATH | XP_003562264.1 | BRADI1G14410.1 | dihydrolipoyllysine-residue succinyltransferase  component of 2-oxoglutarate dehydrogenase complex 1 | dihydrolipoyllysine-residue succinyltransferase component of 2-oxoglutarate dehydrogenase complex 1, mitochondrial-like (438 aa) |
| gi\|475620773\|gb\|EMT31237.1\| | XP_003569582.1 | BRADI2G46840.1 | guanine nucleotide-binding protein subunit  beta-like protein A-like | guanine nucleotide-binding protein subunit beta-like protein A-like (331 aa) |
| gi\|148508784\|gb\|ABQ81648.1\| | XP_003573317.1 | BRADI3G14040.1 | glyceraldehyde-3-phosphate dehydrogenase, cytosolic-like | glyceraldehyde-3-phosphate dehydrogenase, cytosolic-like (337 aa) |
| gi\|125553505\|gb\|EAY99214.1\| | XP_003567810.1 | BRADI2G14840.1 | mitochondrial substrate carrier family protein B-like | mitochondrial substrate carrier family protein B-like (354 aa) |
| gi\|296088147\|emb\|CBI35592.3\| | XP_003580022.1 | BRADI5G14060.1 | pentatricopeptide repeat-containing protein At1g06710, mitochondrial-like | pentatricopeptide repeat-containing protein At1g06710, mitochondrial-like (966 aa) |
| gi\|473993048\|gb\|EMS51931.1\| | XP_003578601.1 | BRADI4G37350.1 | UTP--glucose-1-phosphate uridylyltransferase-like | UTP--glucose-1-phosphate uridylyltransferase-like (475 aa) |
| gi\|294717871\|gb\|ADF31783.1\| | XP_003574727.1 | BRADI3G39630.1 | heat shock protein 81-1 | heat shock protein 81-3-like (699 aa) |
| gi\|474049119\|gb\|EMS53579.1\| | XP_003574727.1 | BRADI3G39630.1 | heat shock protein 81-1 | heat shock protein 81-3-like (699 aa) |
| gi\|223018643\|gb\|ACM78035.1\| | XP_003577785.1 | BRADI4G24367.1 | fructose-bisphosphate aldolase, chloroplastic-like | fructose-bisphosphate aldolase, chloroplastic-like (388 aa) |
| gi\|297322942\|gb\|EFH53363.1\| | XP_003571049.1 | BRADI3G06577.1 | putative multidrug resistance protein-like | putative multidrug resistance protein-like (1242 aa) |
| gi\|473940347\|gb\|EMS50333.1\| | XP_003559884.1 | TRRAP | transformation/transcription domain-associated protein | transformation/transcription domain-associated protein (3884 aa) |
| gi\|474416303\|gb\|EMS67204.1\| | XP_003575382.1 | BRADI3G49070.1 | succinyl-CoA ligase [ADP-forming] subunit beta, mitochondrial-like | succinyl-CoA ligase [ADP-forming] subunit beta, mitochondrial-like (422 aa) |
| gi\|473797438\|gb\|EMS46497.1\| | XP_003573295.1 | BRADI3G13600.1 | K00847 fructokinase [EC-2.7.1.4] | K00847 fructokinase [EC-2.7.1.4] (338 aa) |
| gi\|473846061\|gb\|EMS47382.1\| | XP_003567673.1 | BRADI2G12727.2 | ABC transporter C family member 4-like | ABC transporter C family member 4-like (1526 aa) |
| gi\|2827002\|gb\|AAB99745.1\| | XP_003578898.1 | BRADI4G04220.1 | heat shock cognate 70 kDa protein 2-like | heat shock cognate 70 kDa protein 2-like (648 aa) |
| gi\|474404660\|gb\|EMS66394.1\| | XP_003580734.1 | BRADI5G02160.1 | K01476 arginase [EC-3.5.3.1] | K01476 arginase [EC-3.5.3.1] (342 aa) |
| gi\|474224464\|gb\|EMS59382.1\| | XP_003565155.1 | BRADI2G61940.1 | bifunctional polymyxin resistance protein ArnA-like | bifunctional polymyxin resistance protein ArnA-like (393 aa) |
| gi\|473796898\|gb\|EMS46492.1\| | XP_003565155.1 | BRADI2G61940.1 | bifunctional polymyxin resistance protein ArnA-like | bifunctional polymyxin resistance protein ArnA-like (393 aa) |
| gi\|475560198\|gb\|EMT13510.1\| | XP_003565155.1 | BRADI2G61940.1 | bifunctional polymyxin resistance protein ArnA-like | bifunctional polymyxin resistance protein ArnA-like (393 aa) |
| gi\|223543328\|gb\|EEF44860.1\| | XP_003569013.1 | BRADI2G37470.1 | glutelin type-A 1-like | glutelin type-A 1-like (358 aa) |
| gi\|475481099\|gb\|EMT02862.1\| | XP_003557240.1 | BRADI1G51670.1 | K00615 transketolase [EC-2.2.1.1] | K00615 transketolase [EC-2.2.1.1] (741 aa) |
| gi\|474094791\|gb\|EMS55039.1\| | XP_003557907.1 | BRADI1G61330.1 | peptidyl-prolyl cis-trans isomerase E-like | peptidyl-prolyl cis-trans isomerase E-like (246 aa) |
| gi\|474105890\|gb\|EMS55466.1\| | XP_003567630.1 | BRADI2G12150.2 | S-adenosylmethionine synthase 3-like | Catalyzes the formation of S-adenosylmethionine from methionine and ATP (By similarity) (395 aa) |
| gi\|474103525\|gb\|EMS55348.1\| | XP_003562281.1 | BRADI1G01580.1 | elongation factor Tu, mitochondrial-like | This protein promotes the GTP-dependent binding of aminoacyl-tRNA to the A-site of ribosomes during protein biosynthesis (By similarity) (455 aa) |
| gi\|473933356\|gb\|EMS50043.1\| | XP_003569476.1 | BRADI2G45420.1 | isocitrate dehydrogenase [NADP], chloroplastic-like | isocitrate dehydrogenase [NADP], chloroplastic-like (412 aa) |
| gi\|473825073\|gb\|EMS47104.1\| | XP_003572718.1 | BRADI3G10720.1 | 26S protease regulatory subunit 6B homolog | 26S protease regulatory subunit 6B homolog (419 aa) |
| gi\|5923877\|gb\|AAD56395.1\|AF184059_1 | XP_003567701.1 | BRADI2G13110.1 | probable glutathione S-transferase GSTF1-like | probable glutathione S-transferase GSTF1-like (219 aa) |
| gi\|474215427\|gb\|EMS59019.1\| | XP_003564936.1 | BRADI2G58870.1 | uncharacterized LOC100845167 | uncharacterized LOC100845167 (254 aa) |
| gi\|474213923\|gb\|EMS58988.1\| | XP_003568594.1 | BRADI2G27390.1 | 26S proteasome non-ATPase regulatory subunit 14-like | 26S proteasome non-ATPase regulatory subunit 14-like (306 aa) |
| gi\|119388731\|gb\|ABL74262.1\| | XP_003577716.1 | BRADI4G22630.2 | alcohol dehydrogenase 1-like | alcohol dehydrogenase 1-like (379 aa) |
| gi\|253783729\|emb\|CAZ76054.1\| | XP_003573317.1 | BRADI3G14040.1 | glyceraldehyde-3-phosphate dehydrogenase, cytosolic-like | glyceraldehyde-3-phosphate dehydrogenase, cytosolic-like (337 aa) |
| gi\|4158232\|emb\|CAA77237.1\| | XP_003562308.1 | BRADI1G15050.1 | UDP-arabinopyranose mutase 1-like | UDP-arabinopyranose mutase 1-like (369 aa) |
| gi\|473980005\|gb\|EMS51512.1\| | XP_003567777.1 | BRADI2G14270.1 | notchless protein homolog | notchless protein homolog (471 aa) |
| gi\|474113969\|gb\|EMS55766.1\| | XP_003581066.1 | BRADI5G02890.1 | RuBisCO large subunit-binding protein subunit alpha,  chloroplastic-like | ruBisCO large subunit-binding protein subunit alpha, chloroplastic-like (578 aa) |
| gi\|109150356\|dbj\|BAE96093.1\| | XP_003561832.1 | BRADI1G68450.1 | glucan endo-1,3-beta-glucosidase 10-like | glucan endo-1,3-beta-glucosidase 10-like (431 aa) |
| gi\|46358940\|gb\|AAS88729.1\| | XP_003580229.1 | BRADI5G16900.1 | beta-fructofuranosidase 1-like | beta-fructofuranosidase 1-like (679 aa) |
| gi\|308808452\|ref\|XP_003081536.1\| | XP_003563028.1 | BRADI1G26310.1 | calcium-dependent protein kinase isoform 2-like | calcium-dependent protein kinase isoform 2-like (532 aa) |
| gi\|223526474\|gb\|EEF28747.1\| | XP_003564533.1 | BRADI2G54080.1 | calcium-dependent protein kinase 13-like | calcium-dependent protein kinase 13-like (561 aa) |
| gi\|473895095\|gb\|EMS48917.1\| | XP_003561381.1 | BRADI1G56270.1 | glucan endo-1,3-beta-glucosidase 8-like | glucan endo-1,3-beta-glucosidase 8-like (494 aa) |
| gi\|475532979\|gb\|EMT08314.1\| | XP_003570429.1 | BRADI3G55870.1 | RNA-binding protein Nova-1-like | RNA-binding protein Nova-1-like (349 aa) |
| gi\|474012573\|gb\|EMS52605.1\| | XP_003578898.1 | BRADI4G04220.1 | heat shock cognate 70 kDa protein 2-like | heat shock cognate 70 kDa protein 2-like (648 aa) |
| gi\|58533119\|gb\|AAW78915.1\| | XP_003562930.1 | BRADI1G02580.1 | K10355 actin | other eukaryote (377 aa) |
| gi\|474069724\|gb\|EMS54290.1\| | XP_003564704.1 | BRADI2G05710.1 | mitochondrial-processing peptidase subunit alpha-like | mitochondrial-processing peptidase subunit alpha-like (505 aa) |
| gi\|460393692\|ref\|XP_004242443.1\| | XP_003565059.1 | BRADI2G60660.1 | probable calcium-binding protein CML10-like | probable calcium-binding protein CML10-like (195 aa) |
| gi\|460408572\|ref\|XP_004249717.1\| | XP_003580477.1 | BRADI5G20380.1 | wall-associated receptor kinase 2-like | wall-associated receptor kinase 2-like (737 aa) |
| gi\|528066018\|emb\|CCP19633.1\| | XP_003557467.1 | BRADI1G55260.1 | uncharacterized LOC100829326 | uncharacterized LOC100829326 (248 aa) |
| gi\|460382941\|ref\|XP_004237194.1\| | XP_003560577.1 | BRADI1G35980.1 | cytochrome P450 93A1-like | cytochrome P450 93A1-like (523 aa) |
| gi\|475574945\|gb\|EMT17122.1\| | XP_003573356.1 | BRADI3G01640.1 | UTP--glucose-1-phosphate uridylyltransferase-like | UTP--glucose-1-phosphate uridylyltransferase-like (469 aa) |
| gi\|158701881\|gb\|ABW77317.1\| | XP_003568785.1 | BRADI2G33450.1 | NADP-dependent malic enzyme, chloroplastic-like | NADP-dependent malic enzyme, chloroplastic-like (570 aa) |
| gi\|310619520\|gb\|ADP01875.1\| | XP_003557139.1 | BRADI1G50090.1 | granule-bound starch synthase 1, chloroplastic/amyloplastic-like | granule-bound starch synthase 1, chloroplastic/amyloplastic-like (603 aa) |
| gi\|474139599\|gb\|EMS56480.1\| | XP_003562501.1 | BRADI1G18650.1 | 4-alpha-glucanotransferase DPE2-like | 4-alpha-glucanotransferase DPE2-like (929 aa) |
| gi\|332660383\|gb\|AEE85783.1\| | XP_003574123.1 | BRADI3G29900.1 | 1-acyl-sn-glycerol-3-phosphate acyltransferase 1, chloroplastic-like | 1-acyl-sn-glycerol-3-phosphate acyltransferase 1, chloroplastic-like (332 aa) |
| gi\|32400818\|gb\|AAP80641.1\|AF475120_1 | XP_003563677.1 | BRADI1G36830.1 | 26S protease regulatory subunit 8 homolog A-like | 26S protease regulatory subunit 8 homolog A-like (422 aa) |
| gi\|473843112\|gb\|EMS47369.1\| | XP_003573112.1 | BRADI3G59660.1 | peroxidase 65-like | peroxidase 65-like (349 aa) |
| gi\|474446079\|gb\|EMS68621.1\| | XP_003573873.1 | BRADI3G24680.1 | vacuolar-sorting receptor 3-like | acuolar-sorting receptor 3-like (629 aa) |
| gi\|30688675\|ref\|NP_850351.1\| | XP_003558094.1 | BRADI1G64660.1 | uncharacterized LOC100838122 | uncharacterized LOC100838122 (612 aa) |
| gi\|473813413\|gb\|EMS46838.1\| | XP_003558025.1 | BRADI1G63920.1 | uncharacterized LOC100843292 | uncharacterized LOC100843292 (787 aa) |
| ODO2B_ARATH | XP_003558524.1 | BRADI1G70450.1 | pre-mRNA-processing factor 39-like | pre-mRNA-processing factor 39-like (724 aa) |
| PS17_PINST | XP_003564357.1 | GI | protein GIGANTEA-like | protein GIGANTEA-like (1155 aa) |
| gi\|474343757 | XP_003558836.1 | BRADI1G75590.1 | ABC transporter C family member 5-like | ABC transporter C family member 5-like (1505 aa) |
| RR16_CYAPA | XP_003570301.1 | BRADI3G54140.1 | phosphoinositide 3-kinase regulatory subunit 4-like | phosphoinositide 3-kinase regulatory subunit 4-like (1450 aa) |
| RF1_DESAD | XP_003568273.1 | BRADI2G22820.1 | putative respiratory burst oxidase homolog protein H-like | putative respiratory burst oxidase homolog protein H-like (835 aa) |
| NRDR_DECAR | XP_003558600.1 | BRADI1G71530.1 | LL-diaminopimelate aminotransferase, chloroplastic-like | LL-diaminopimelate aminotransferase, chloroplastic-like (437 aa) |
| gi\|259662485 | XP_003574154.1 | BRADI3G03040.1 | proteasome subunit alpha type-1-like | The proteasome is a multicatalytic proteinase complex which is characterized by its ability to cleave peptides with Arg, Phe, Tyr, Leu, and Glu adjacent to the leaving group at neutral or slightly basic pH. The proteasome has an ATP-dependent proteolytic activity (By similarity) (283 aa) |

**Table S2b. Protein interaction network analysis by searching the STRING 10.0 according to TAIR homologous proteins.**

| **Accession number** | **TAIR homologous proteins** | **STRING Name** | **Protein Name** | **Description** |
| --- | --- | --- | --- | --- |
| gi\|473881465\|gb\|EMS47979.1\| | NP_191835.1 | ATGSTF13 | Glutathione S-transferase-like protein | May be involved in the conjugation of reduced glutathione to a wide number of exogenous and endogenous hydrophobic electrophiles and have a detoxification role against certain herbicides (By similarity) (219 aa) |
| gi\|226897531\|gb\|ACO90195.1\| | NP_001119245.1 | CSD3 | copper/zinc superoxide dismutase 3 | Destroys radicals which are normally produced within the cells and which are toxic to biological systems (By similarity) (164 aa) |
| gi\|109892850\|sp\|P84733.1\|PS17_PINST | NP_187475.1 | AT3G08630 | uncharacterized protein | uncharacterized protein (339 aa) |
| gi\|473822195\|gb\|EMS47048.1\| | NP_195511.1 | ELI3-1 | elicitor-activated gene 3-1 | Involved in lignin biosynthesis. Catalyzes the final step specific for the production of lignin monomers. Catalyzes the NADPH-dependent reduction of coniferaldehyde, 5- hydroxyconiferaldehyde, sinapaldehyde, 4-coumaraldehyde and caffeyl aldehyde to their respective alcohols (357 aa) |
| gi\|474042704\|gb\|EMS53325.1\| | NP_186872.1 | RGP1 | reversibly glycosylated polypeptide 1 | UDP-L-arabinose mutase involved in the biosynthesis of cell wall non-cellulosic polysaccharides. Catalyzes the interconvertion of UDP-L-arabinopyranose (UDP-Arap) and UDP-L- arabinofuranose (UDP-Araf) in vitro. Preferentially catalyzes the formation of UDP-Arap from UDP-Araf. At thermodynamic equilibrium in vitro the ratio of the pyranose form over the furanose form is 95-5. Is not active on other UDP-sugars (UDP-Gal, UDP-Xyl, UDP- Glc, GDP-Man and GDP-Fuc). Functions redundantly with RGP2 and is essential for proper cell walls and pollen developm [...] (357 aa) |
| gi\|4098323\|gb\|AAD10489.1\| | NP_568959.1 | TUB2 | tubulin beta chain 2 | Tubulin is the major constituent of microtubules. It binds two moles of GTP, one at an exchangeable site on the beta chain and one at a non-exchangeable site on the alpha chain (450 aa) |
| gi\|226897531\|gb\|ACO90195.1\| | NP_001119245.1 | CSD3 | copper/zinc superoxide dismutase 3 | Destroys radicals which are normally produced within the cells and which are toxic to biological systems (By similarity) (164 aa) |
| gi\|3393062\|emb\|CAA76758.1\| | NP_195899.1 | GSTL3 | Glutathione transferase L3 | Catalyzes the glutathione-dependent reduction of S- glutathionylquercetin to quercetin (235 aa) |
| gi\|19912725\|dbj\|BAB88645.1\| | AAB49302.1 | AOX1A | alternative oxidase 1A | Catalyzes the cyanide-resistant oxidation of ubiquinol and the reduction of molecular oxygen to water, but does not translocate protons and consequently is not linked to oxidative phosphorylation. Increases respiration when the cytochrome respiratory pathway is restricted, or in response to low temperatures (354 aa) |
| gi\|91208366\|sp\|Q47IH0.1\|NRDR_DECAR | [NP_001190702.1](https://www.ncbi.nlm.nih.gov/protein/334186444?report=genbank&log$=protalign&blast_rank=1&RID=4HE9MCZA01R) | PGRL1B | PGR5-like B | Ferredoxin-plastoquinone reductase involved in cyclic electron flow (CEF) around photosystem I. The homodimer is probably not involved in CEF (313 aa) |
| gi\|474072613 | NP_180869.1 | CSLD1 | cellulose synthase-like D1 | Thought to be a Golgi-localized beta-glycan synthase that polymerize the backbones of noncellulosic polysaccharides (hemicelluloses) of plant cell wall (1036 aa) |
| gi\|482576126\|gb\|EOA40313.1 | NP_850940.1 | MGP2 | MALE GAMETOPHYTE DEFECTIVE 2 | MALE GAMETOPHYTE DEFECTIVE 2 (474 aa) |
| gi\|475561248\|gb\|EMT13737.1\| | NP_194193.2 | PGI1 | phosphoglucose isomerase 1 | Promotes the synthesis of starch in leaves (613 aa) |
| gi\|475493180\|gb\|EMT03692.1\| | NP_200879.1 | AT5G60710 | C3H4 type zinc finger protein | C3H4 type zinc finger protein (704 aa) |
| gi\|474036467\|gb\|EMS53132.1\| | AAM98163.1 | CHLI2 | magnesium chelatase i2 | Involved in chlorophyll biosynthesis. Catalyzes the insertion of magnesium ion into protoporphyrin IX to yield Mg- protoporphyrin IX. The reaction takes place in two steps, with an ATP-dependent activation followed by an ATP-dependent chelation step. Possesses low affinity for ATP and may play a limited role in chlorophyll biosynthesis, and contributes to the assembly of the Mg-chelatase complex (418 aa) |
| gi\|474368068\|gb\|EMS63646.1\| | AAM64988.1 | MVD1 | mevalonate diphosphate decarboxylase 1 | mevalonate diphosphate decarboxylase 1 (412 aa) |
| gi\|474286973\|gb\|EMS61151.1\| | NP_565447.1 | SLP3 | subtilisin-like serine protease 3 | subtilisin-like serine protease 3 (815 aa) |
| gi\|449445262\|ref\|XP_004140392.1\| | NP_565280.1 | AT2G02050 | putative NADH-ubiquinone oxidoreductase B18 subunit | Accessory subunit of the mitochondrial membrane respiratory chain NADH dehydrogenase (Complex I), that is believed not to be involved in catalysis. Complex I functions in the transfer of electrons from NADH to the respiratory chain. The immediate electron acceptor for the enzyme is believed to be ubiquinone (By similarity) (103 aa) |
| gi\|475619612\|gb\|EMT30822.1\| | NP_566469.1 | EIF4A1 | translational initiation factor 4A-1 | ATP-dependent RNA helicase which is a subunit of the eIF4F complex involved in cap recognition and is required for mRNA binding to ribosome. In the current model of translation initiation, eIF4A unwinds RNA secondary structures in the 5’-UTR of mRNAs which is necessary to allow efficient binding of the small ribosomal subunit, and subsequent scanning for the initiator codon (415 aa) |
| gi\|475620279\|gb\|EMT31062.1\| | CAA52019.1 | FAR2 | MALE STERILITY 2 | Catalyzes the reduction of fatty acyl-CoA to fatty alcohols. Involved in the synthesis of the lipid component in sporopollenin (616 aa) |
| gi\|335906213\|gb\|AEH68230.1\| | NP_173594.1 | PDIL1-1 | PDI-like 1-1 | Protein disulfide isomerase that associates with RD21A protease for trafficking from the ER through the Golgi to lytic and protein storage vacuoles of endothelial cells in developing seeds. Regulates the timing of programmed cell death (PCD) of the endothelial cells by chaperoning and inhibiting cysteine proteases during their trafficking to vacuoles (501 aa) |
| gi\|525778513\|ref\|YP_008239101.1\| | NP_051067.1 | RBCL | ribulose-bisphosphate carboxylases.[Source-TAIR;Acc-ATCG00490] | RuBisCO catalyzes two reactions- the carboxylation of D- ribulose 1,5-bisphosphate, the primary event in carbon dioxide fixation, as well as the oxidative fragmentation of the pentose substrate in the photorespiration process. Both reactions occur simultaneously and in competition at the same active site (By similarity) (479 aa) |
| gi\|474152910\|gb\|EMS57002.1\| | AAY96720.1 | LAP6 | LESS ADHESIVE POLLEN 6 | LESS ADHESIVE POLLEN 6 (395 aa) |
| gi\|473821746\|gb\|EMS47029.1\| | NP_568114.1 | AtCDC48C | cell division cycle 48C | Probably functions in cell division and growth processes. Interacts with certain SNAREs as part of specialized membrane fusion events where vesicles from the same organelle fuse (homotypic fusion) (By similarity) (810 aa) |
| gi\|1350876\|sp\|P48139.1\|RR16_CYAPA | NP_051041.1 | RPS16 | ribosomal protein S16 | ribosomal protein S16 (79 aa) |
| gi\|259585219\|sp\|C6BYQ4.1\|RF1_DESAD | NP_191850.2 | APG3 | ALBINO AND PALE GREEN | ALBINO AND PALE GREEN (422 aa) |
| gi\|474305481\|gb\|EMS61741.1\| | NP_565001.1 | AT1G70790 | calcium-dependent lipid-binding domain-containing protein | calcium-dependent lipid-binding domain-containing protein (185 aa) |
| gi\|22330270\|ref\|NP_175981.2\| | NP_175981.2 | CSLE1 | cellulose synthase like E1 | Thought to be a Golgi-localized beta-glycan synthase that polymerize the backbones of noncellulosic polysaccharides (hemicelluloses) of plant cell wall (729 aa) |
| gi\|23504741\|emb\|CAD29476.1\| | NP_191835.1 | ATGSTF13 | Glutathione S-transferase-like protein; | May be involved in the conjugation of reduced glutathione to a wide number of exogenous and endogenous hydrophobic electrophiles and have a detoxification role against certain herbicides (By similarity) (219 aa) |
| gi\|308197130\|sp\|Q8H107.2\|ODO2B_ARATH | NP_567761.1 | AT4G26910 | dihydrolipoyllysine-residue succinyltransferase component of 2-oxoglutarate dehydrogenase complex 2 | The 2-oxoglutarate dehydrogenase complex catalyzes the overall conversion of 2-oxoglutarate to succinyl-CoA and CO(2). It contains multiple copies of three enzymatic components- 2- oxoglutarate dehydrogenase (E1), dihydrolipoamide succinyltransferase (E2) and lipoamide dehydrogenase (E3) (By similarity) (464 aa) |
| gi\|475620773\|gb\|EMT31237.1\| | NP_173248.1 | ATARCA | guanine nucleotide-binding protein subunit beta-like protein | Major component of the RACK1 regulatory proteins that play a role in multiple signal transduction pathways. Involved in multiple hormone responses and developmental processes (327 aa) |
| gi\|77552263\|gb\|ABA95060.1\| | NP_199366.1 | AT5G45530 | uncharacterized protein | uncharacterized protein (798 aa) |
| gi\|148508784\|gb\|ABQ81648.1\| | NP_187062.1 | GAPC1 | glyceraldehyde-3-phosphate dehydrogenase, cytosolic | Key enzyme in glycolysis that catalyzes the first step of the pathway by converting D-glyceraldehyde 3-phosphate (G3P) into 3-phospho-D-glyceroyl phosphate. Essential for the maintenance of cellular ATP levels and carbohydrate metabolism. Involved in response to oxidative stress by mediating plant responses to abscisic acid (ABA) and water deficits through the activation of PLDDELTA and production of phosphatidic acid (PA), a multifunctional stress signaling lipid in plants. Required for full fertility. Binds DNA in vitro (338 aa) |
| gi\|125553505\|gb\|EAY99214.1\| | NP_192019.1 | ADNT1 | adenine nucleotide transporter 1 | Mitochondrial adenylate carrier that catalyzes specifically the transport of ATP, ADP and AMP by a counter- exchange mechanism across the inner mitochondrial membrane. Substrate preference in reconstituted proteoliposomes is ATP > AMP > ADP. May play a role in oxidative phosphorylation and be important for the provision of energy required to support growth in heterotrophic tissues (366 aa) |
| gi\|296088147\|emb\|CBI35592.3\| | NP_187385.1 | AT3G07290 | pentatricopeptide repeat-containing protein | pentatricopeptide repeat-containing protein (880 aa) |
| gi\|473993048\|gb\|EMS51931.1\| | NP_186975.1 | UGP1 | UDP-GLUCOSE PYROPHOSPHORYLASE 1 | Plays a central role as a glucosyl donor in cellular metabolic pathways (By similarity) (469 aa) |
| gi\|294717871\|gb\|ADF31783.1\| | NP_200411.1 | Hsp81.4 | HEAT SHOCK PROTEIN 81.4 | Molecular chaperone. Due to its association with certain proteins such as hormone receptors and some classes of kinases, it is implicated in signal transduction and development. Has ATPase activity (By similarity) (699 aa) |
| gi\|474049119\|gb\|EMS53579.1\| | NP_200411.1 | Hsp81.4 | HEAT SHOCK PROTEIN 81.4 | Molecular chaperone. Due to its association with certain proteins such as hormone receptors and some classes of kinases, it is implicated in signal transduction and development. Has ATPase activity (By similarity) (699 aa) |
| gi\|223018643\|gb\|ACM78035.1\| | NP_568049.1 | FBA2 | fructose-bisphosphate aldolase 2 | fructose-bisphosphate aldolase 2 (398 aa) |
| gi\|297322942\|gb\|EFH53363.1\| | NP_189480.1 | ABCB18 | ATP-binding cassette B18 | ATP-binding cassette B18 (1225 aa) |
| gi\|473940347\|gb\|EMS50333.1\| | NP_179383.3 | AT2G17930 | transformation/transcription domain-associated protein | transformation/transcription domain-associated protein (3858 aa) |
| gi\|474416303\|gb\|EMS67204.1\| | NP_179632.1 | AT2G20420 | Succinyl-CoA ligase [GDP-forming] subunit beta | Succinyl-CoA ligase [GDP-forming] subunit beta (421 aa) |
| gi\|77554581\|gb\|ABA97377.1\| | AAF67380.1 |  |  |  |
| gi\|473797438\|gb\|EMS46497.1\| | NP_192764.1 | AT4G10260 | fructokinase | May play an important role in maintaining the flux of carbon towards starch formation (By similarity) (324 aa) |
| gi\|473846061\|gb\|EMS47382.1\| | CAB86942.1 | ABCC10 | ATP-binding cassette C10 | Pump for glutathione S-conjugates (By similarity) (1453 aa) |
| gi\|2827002\|gb\|AAB99745.1\| | NP_187864.1 | HSP70 | heat shock protein 70 | Component of the Mediator complex, a coactivator involved in the regulated transcription of nearly all RNA polymerase II-dependent genes. Mediator functions as a bridge to convey information from gene-specific regulatory proteins to the basal RNA polymerase II transcription machinery. The Mediator complex, having a compact conformation in its free form, is recruited to promoters by direct interactions with regulatory proteins and serves for the assembly of a functional preinitiation complex with RNA polymerase II and the general transcription factors (By similarity) (650 aa) |
| gi\|474404660\|gb\|EMS66394.1\| | NP_192629.1 | ARGAH1 | arginase | arginase (342 aa) |
| gi\|474224464\|gb\|EMS59382.1\| | NP_563807.1 | AXS2 | UDP-apiose/xylose synthase | UDP-apiose/xylose synthase (389 aa) |
| gi\|473796898\|gb\|EMS46492.1\| | NP_172669.2 | AT1G12050 | fumarylacetoacetase | fumarylacetoacetase (421 aa) |
| gi\|475560198\|gb\|EMT13510.1\| | NP_172255.1 | AT1G07750 | cupin domain-containing protein | cupin domain-containing protein (356 aa) |
| gi\|223543328\|gb\|EEF44860.1\| | NP_001330641.1 |  |  |  |
| gi\|475481099\|gb\|EMT02862.1\| | NP_567103.1 | AT3G60750 | Transketolase | Catalyzes the reversible transfer of a two-carbon ketol group from fructose-6-phosphate or sedoheptulose-7-phosphate to glyceraldehyde-3-phosphate to yield xylulose-5-phosphate and erythrose-4-phosphate or ribose-5-phosphate, respectively (By similarity). Could act as a stress sensor involved in adaptation process (741 aa) |
| gi\|474094791\|gb\|EMS55039.1\| | NP_567021.1 | SCL30 | SC35-like splicing factor 30 | SC35-like splicing factor 30 (262 aa) |
| gi\|474105890\|gb\|EMS55466.1\| | NP_192094.1 | SAM-2 | S-adenosylmethionine synthase 2 | Catalyzes the formation of S-adenosylmethionine from methionine and ATP. The overall synthetic reaction is composed of two sequential steps, AdoMet formation and the subsequent tripolyphosphate hydrolysis which occurs prior to release of AdoMet from the enzyme (By similarity) (393 aa) |
| gi\|18076790\|emb\|CAC85913.1\| | NP_001154464.1 | PGM2 | phosphoglucomutase | This enzyme participates in both the breakdown and synthesis of glucose (By similarity) (662 aa) |
| gi\|474103525\|gb\|EMS55348.1\| | NP_192202.1 | AT4G02930 | Elongation factor Tu | This protein promotes the GTP-dependent binding of aminoacyl-tRNA to the A-site of ribosomes during protein biosynthesis (By similarity) (454 aa) |
| gi\|473933356\|gb\|EMS50043.1\| | NP_176768.1 | cICDH | isocitrate dehydrogenase | May supply 2-oxoglutarate for amino acid biosynthesis and ammonia assimilation via the glutamine synthetase/glutamate synthase (GS/GOGAT) pathway. May be involved in the production of NADPH to promote redox signaling or homeostasis in response to oxidative stress (410 aa) |
| gi\|473825073\|gb\|EMS47104.1\| | AAF22522.1 | RPT2a | regulatory particle AAA-ATPase 2A | The 26S protease is involved in the ATP-dependent degradation of ubiquitinated proteins. The regulatory (or ATPase) complex confers ATP dependency and substrate specificity to the 26S complex. Is required for the maintenance of postembryonic root and shoot meristems. Has a specific role in the regulation of organs size (443 aa) |
| gi\|5923877\|gb\|AAD56395.1\|AF184059_1 | NP_200637.1 | RPT3 | regulatory particle triple-A ATPase 3 | The 26S protease is involved in the ATP-dependent degradation of ubiquitinated proteins. The regulatory (or ATPase) complex confers ATP dependency and substrate specificity to the 26S complex (408 aa) |
| gi\|474215427\|gb\|EMS59019.1\| | NP_187376.1 | AT3G07200 | RING/U-box domain-containing protein | RING/U-box domain-containing protein (182 aa) |
| gi\|474213923\|gb\|EMS58988.1\| | AAM64349.1 | AT5G23540 | 26S proteasome non-ATPase regulatory subunit 14 | Metalloprotease component of the 26S proteasome that specifically cleaves ’Lys-63’-linked polyubiquitin chains. The 26S proteasome is involved in the ATP-dependent degradation of ubiquitinated proteins. The function of the ’Lys-63’-specific deubiquitination of the proteasome is unclear (By similarity) (308 aa) |
| gi\|119388731\|gb\|ABL74262.1\| | AAF23554.1 | ADH1 | alcohol dehydrogenase 1 | alcohol dehydrogenase 1 (379 aa) |
| gi\|253783729\|emb\|CAZ76054.1\| | NP_172801.1 | GAPC2 | glyceraldehyde 3-phosphate dehydrogenase | Key enzyme in glycolysis that catalyzes the first step of the pathway by converting D-glyceraldehyde 3-phosphate (G3P) into 3-phospho-D-glyceroyl phosphate. Essential for the maintenance of cellular ATP levels and carbohydrate metabolism (By similarity). Binds DNA in vitro (338 aa) |
| gi\|4158232\|emb\|CAA77237.1\| | NP_197069.1 | RGP2 | reversibly glycosylated polypeptide 2 | UDP-L-arabinose mutase involved in the biosynthesis of cell wall non-cellulosic polysaccharides. Catalyzes the interconvertion of UDP-L-arabinopyranose (UDP-Arap) and UDP-L- arabinofuranose (UDP-Araf) in vitro. Preferentially catalyzes the formation of UDP-Arap from UDP-Araf. At thermodynamic equilibrium in vitro the ratio of the pyranose form over the furanose form is 95-5. Is not active on other UDP-sugars (UDP-Gal, UDP-Xyl, UDP- Glc, GDP-Man and GDP-Fuc). Functions redundantly with RGP2 and is essential for proper cell walls and pollen developm [...] (360 aa) |
| gi\|473980005\|gb\|EMS51512.1\| | NP_188434.2 | AT3G18060 | transducin/WD40 domain-containing protein | transducin/WD40 domain-containing protein (609 aa) |
| gi\|474113969\|gb\|EMS55766.1\| | NP_180367.1 | CPN60A | chaperonin-60alpha | Binds RuBisCO small and large subunits and is implicated in the assembly of the enzyme oligomer. Involved in protein assisted folding. Required for proper chloroplast development (586 aa) |
| gi\|109150356\|dbj\|BAE96093.1\| | AAM63339.1 | BGL2 | beta-1,3-glucanase 2 | Implicated in the defense of plants against pathogens (339 aa) |
| gi\|46358940\|gb\|AAS88729.1\| | NP_564798.1 | AT1G62660 | beta-fructofuranosidase | Possible role in the continued mobilization of sucrose to sink organs (By similarity) (648 aa) |
| gi\|22330325\|ref\|NP_683450.1\| | NP_683450.1 | AT1G59690 | F-box associated ubiquitination effector family protein | F-box associated ubiquitination effector family protein (143 aa) |
| gi\|308808452\|ref\|XP_003081536.1\| | NP_181425.1 | CPK20 | calcium-dependent protein kinase 20 | May play a role in signal transduction pathways that involve calcium as a second messenger (583 aa) |
| gi\|223526474\|gb\|EEF28747.1\| | [NP_190753.2](https://www.ncbi.nlm.nih.gov/protein/22331739?report=genbank&log$=protalign&blast_rank=1&RID=4JFJF0YY015) | CPK13 | calcium-dependent protein kinase 13 | May play a role in signal transduction pathways that involve calcium as a second messenger (528 aa) |
| gi\|473895095\|gb\|EMS48917.1\| | NP_193361.4 | AT4G16260 | catalytic/ cation binding / hydrolase | catalytic/ cation binding / hydrolase (344 aa) |
| gi\|475532979\|gb\|EMT08314.1\| | NP_175569.1 | AT4G16260 | catalytic/ cation binding / hydrolase | catalytic/ cation binding / hydrolase (344 aa) |
| gi\|474012573\|gb\|EMS52605.1\| | NP_195870.1 | HSC70-1 | heat shock 70kDa protein 1/8 | Component of the Mediator complex, a coactivator involved in the regulated transcription of nearly all RNA polymerase II-dependent genes. Mediator functions as a bridge to convey information from gene-specific regulatory proteins to the basal RNA polymerase II transcription machinery. The Mediator complex, having a compact conformation in its free form, is recruited to promoters by direct interactions with regulatory proteins and serves for the assembly of a functional preinitiation complex with RNA polymerase II and the general transcription factors (By s [...] (651 aa) |
| gi\|58533119\|gb\|AAW78915.1\| | NP_196543.1 | ACT7 | actin 7 | Actins are highly conserved proteins that are involved in various types of cell motility and are ubiquitously expressed in all eukaryotic cells. Essential component of cell cytoskeleton; plays an important role in cytoplasmic streaming, cell shape determination, cell division, organelle movement and extension growth. This is considered as one of the vegetative actins which is involved in the regulation of hormone-induced plant cell proliferation and callus formation (377 aa) |
| gi\|474069724\|gb\|EMS54290.1\| | NP_566548.1 | MPPalpha | mitochondrial processing peptidase | Cleaves presequences (transit peptides) from mitochondrial protein precursors (By similarity) (499 aa) |
| gi\|460393692\|ref\|XP_004242443.1\| | NP_172089.1 | RHS2 | calmodulin-like protein 7 | Potential calcium sensor (By similarity) (150 aa) |
| gi\|460408572\|ref\|XP_004249717.1\| | NP_173546.1 | WAK5 | wall associated kinase 5 | Serine/threonine-protein kinase that may function as a signaling receptor of extracellular matrix component. Binding to pectin may have significance in the control of cell expansion, morphogenesis and development (733 aa) |
| gi\|528066018\|emb\|CCP19633.1\| | NP_180620.1 | AT2G30620 | histone H1.2 | Histones H1 are necessary for the condensation of nucleosome chains into higher-order structures (273 aa) |
| gi\|460382941\|ref\|XP_004237194.1\| | NP_194925.1 | CYP82C2 | cytochrome P450, family 82, subfamily C, polypeptide 2 | Can hydroxylate 8-methoxypsoralen to form 5-hydroxy-8- methoxypsoralen in vivo and in vitro (523 aa) |
| gi\|475574945\|gb\|EMT17122.1\| | NP_197233.1 | UGP2 | UDP-glucose pyrophosphorylase 2 | Plays a central role as a glucosyl donor in cellular metabolic pathways (By similarity) (470 aa) |
| gi\|158701881\|gb\|ABW77317.1\| | NP_197960.1 | NADP-ME3 | NADP-malic enzyme 3 | NADP-malic enzyme 3 (588 aa) |
| gi\|310619520\|gb\|ADP01875.1\| | NP_174566.1 | GBSS1 | granule-bound starch synthase | Required for the synthesis of amylose in endosperm (By similarity) (610 aa) |
| gi\|474139599\|gb\|EMS56480.1\| | NP_181616.3 | DPE2 | 4-alpha-glucanotransferase | Cytosolic alpha-glucanotransferase essential for the cytosolic metabolism of maltose, an intermediate on the pathway by which starch is converted to sucrose in leaves at night. Metabolizes maltose exported from the chloroplast and is specific for beta-maltose. May play a role in freezing tolerance. Temperature drop induces inactivation of DPE2 that leads to rapid accumulation of maltose, a solute that protects cells from freezing damage (955 aa) |
| gi\|332660383\|gb\|AEE85783.1\| | NP_194787.2 | ATS2 | 1-acyl-sn-glycerol-3-phosphate acyltransferase 1 | Converts lysophosphatidic acid (LPA) into phosphatidic acid by incorporating acyl moiety at the 2 position. Has preference for C-16-CoA substrates compared to C-18-CoA substrates. Essential for embryo development during the transition from the globular to the heart stage when chloroplasts begin to form (356 aa) |
| gi\|32400818\|gb\|AAP80641.1\|AF475120_1 | AAF22526.1 | RPT6A | regulatory particle triple-A ATPase 6A | The 26S protease is involved in the ATP-dependent degradation of ubiquitinated proteins. The regulatory (or ATPase) complex confers ATP dependency and substrate specificity to the 26S complex (419 aa) |
| gi\|473843112\|gb\|EMS47369.1\| | BAB10239.1 | AT5G47000 | peroxidase 65 | Removal of H(2)O(2), oxidation of toxic reductants, biosynthesis and degradation of lignin, suberization, auxin catabolism, response to environmental stresses such as wounding, pathogen attack and oxidative stress. These functions might be dependent on each isozyme/isoform in each plant tissue (334 aa) |
| gi\|474446079\|gb\|EMS68621.1\| | NP_179079.1 | VSR4 | vacuolar sorting receptor 4 | Vacuolar-sorting receptor (VSR) involved in clathrin- coated vesicles sorting from Golgi apparatus to vacuoles (By similarity) (628 aa) |
| gi\|30688675\|ref\|NP_850351.1\| | NP_850351.1 | TPR15 | tetratricopeptide repeat 15 | tetratricopeptide repeat 15 (1108 aa) |
| gi\|473813413\|gb\|EMS46838.1\| | AAB70407.1 | TPR15 | tetratricopeptide repeat 15 | tetratricopeptide repeat 15 (1108 aa) |
| ODO2B_ARATH | [NP_567761.1](https://www.ncbi.nlm.nih.gov/protein/18416889?report=genbank&log$=protalign&blast_rank=1&RID=4M0HU8ZX014) | AT4G26910 | dihydrolipoyllysine-residue succinyltransferase component of 2-oxoglutarate dehydrogenase complex 2 | The 2-oxoglutarate dehydrogenase complex catalyzes the overall conversion of 2-oxoglutarate to succinyl-CoA and CO(2). It contains multiple copies of three enzymatic components- 2- oxoglutarate dehydrogenase (E1), dihydrolipoamide succinyltransferase (E2) and lipoamide dehydrogenase (E3) (By similarity) (464 aa) |
| PS17_PINST | NP_187475.1 | AT3G08630 | uncharacterized protein | uncharacterized protein (339 aa) |
| gi\|474343757 | NP_171908.1 | ABCC5 | ATP-binding cassette C5 | Pump for glutathione S-conjugates. Involved in regulation of K(+) and Na(+) cell content. Mediates resistance to NaCl and Li(+), confers sensitivity to sulfonylurea drugs such as glibenclamide (inducer of stomatal opening), and required for stomatal opening regulation by auxin, abscisic acid (ABA) and external Ca(2+). Transports oestradiol-17-(beta-D-glucuronide) (E(2)17G). Involved in the root auxin content regulation that controls the transition from primary root elongation to lateral root formation. Plays a role in ABA-mediated germination inhibition (1514 aa) |
| RR16_CYAPA | NP_051041.1 | RPS16 | ribosomal protein S16 | ribosomal protein S16 (79 aa) |
| RF1_DESAD | [NP_191473.2](https://www.ncbi.nlm.nih.gov/protein/22331862?report=genbank&log$=prottop&blast_rank=7&RID=4M3C0YSN015) | ABCC10 | ATP-binding cassette C10 | Pump for glutathione S-conjugates (By similarity) (1453 aa) |
| NRDR_DECAR | NP_001118430.1 | AT2G32160 | N2227-like domain-containing protein | N2227-like domain-containing protein (463 aa) |
| gi\|259662485 | NP_199093.1 | PAF1 | proteasome alpha subunit F1 | The proteasome is a multicatalytic proteinase complex which is characterized by its ability to cleave peptides with Arg, Phe, Tyr, Leu, and Glu adjacent to the leaving group at neutral or slightly basic pH. The proteasome has an ATP-dependent proteolytic activity. Negatively regulates thiol biosynthesis and arsenic tolerance (278 aa) |
