# Supplementary material for: Chemical hybridizing agent SQ-1-induced male sterility in Triticum aestivum L.: a comparative analysis of the anther proteome
Source: BMC Plant Biol. 2018 Jan 5;18:7. doi: 10.1186/s12870-017-1225-x (PMC5755283; doi:10.1186/s12870-017-1225-x)
Supplement: Supplementary file 6 — Protein interaction network analysis by searching the STRING 10.0 (TAIR homologous proteins). (DOCX 1653 kb) [file 12870_2017_1225_MOESM6_ESM.docx]

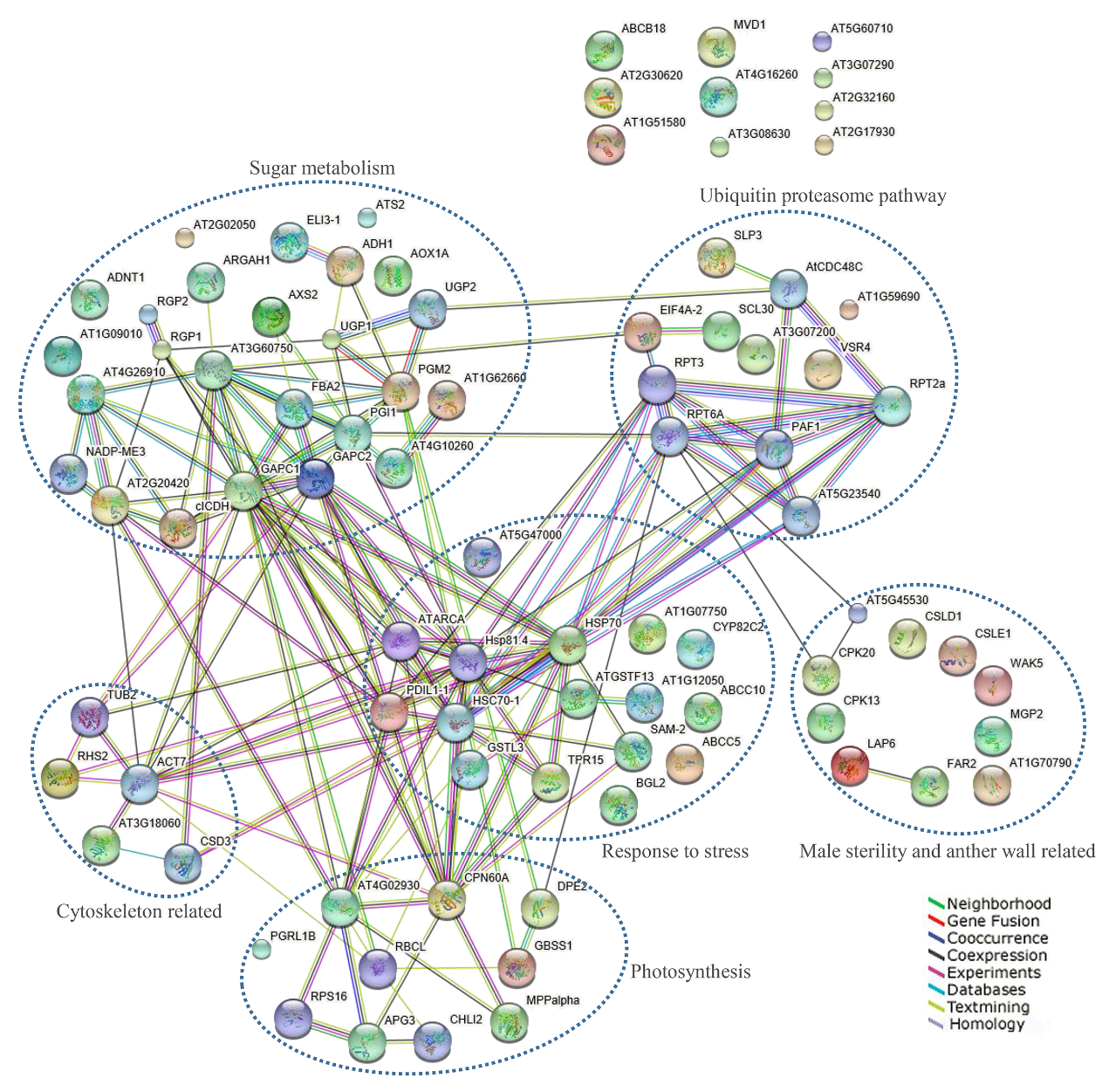


**Figure S4. Protein interaction network analysis by searching the STRING 10.0.**TAIR homologous proteins from DEPs were mapped by searching the STRING 10.0 (http://string-db.org) with a confidence cutoff of 0.4. Colored lines between the proteins indicate the various types of interaction evidence. The details of all the protein nodes were listed in Table S2a-b.
