# Supplementary material for: Chemical hybridizing agent SQ-1-induced male sterility in Triticum aestivum L.: a comparative analysis of the anther proteome
Source: BMC Plant Biol. 2018 Jan 5;18:7. doi: 10.1186/s12870-017-1225-x (PMC5755283; doi:10.1186/s12870-017-1225-x)
Supplement: Supplementary file 7 — Cellular component, molecular function and biological process networks generated by BiNGO. (DOCX 417 kb) [file 12870_2017_1225_MOESM7_ESM.docx]

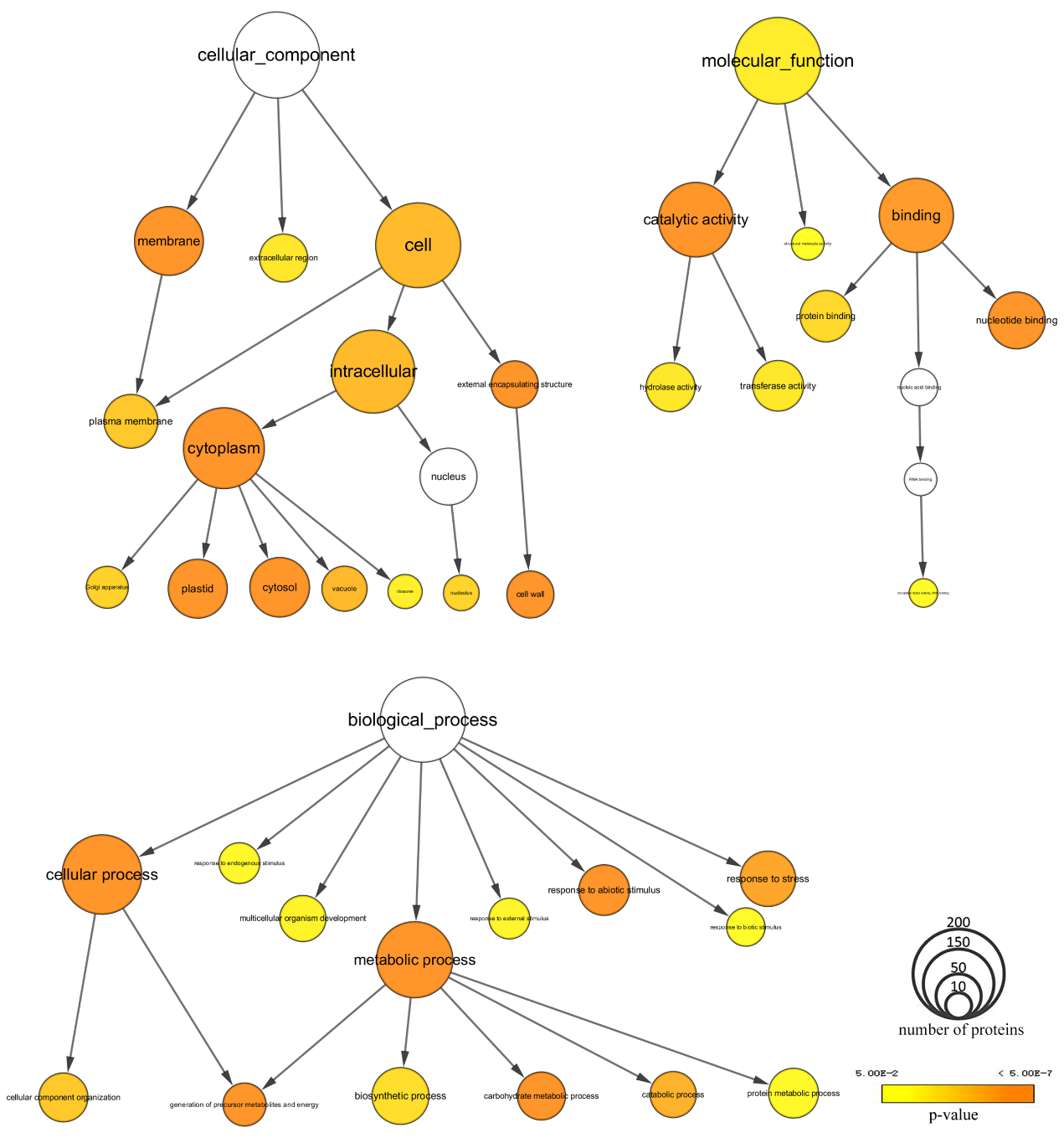


**Figure S5. Cellular component, molecular function and biological process networks** **generated by BiNGO.** Networks generated by the Cytoscape plug-in BiNGO for visualizing GO categories as a network that representation of gene ontologies of homologous proteins. Nodes correspond to GO categories are sized according to the number of proteins. Node colors from yellow to orange denotes the p-value of a hypergeometric test for each enriched GO term. White nodes are not significantly enriched among proteins.
