# Supplementary material for: Chemical hybridizing agent SQ-1-induced male sterility in Triticum aestivum L.: a comparative analysis of the anther proteome
Source: BMC Plant Biol. 2018 Jan 5;18:7. doi: 10.1186/s12870-017-1225-x (PMC5755283; doi:10.1186/s12870-017-1225-x)
Supplement: Supplementary file 8 — BINGO analysis of differentially expressed proteins. (DOCX 20 kb) [file 12870_2017_1225_MOESM8_ESM.docx]

**Table S3. BINGO analysis of differentially expressed proteins.**

| Discarded evidence codes : Overrepresentation | | | | | | |
| --- | --- | --- | --- | --- | --- | --- |
| Selected statistical test : Hypergeometric test | | | | | | |
| Selected correction : Benjamini & Hochberg False Discovery Rate (FDR) correction | | | | | | |
| Selected significance level : 0.05 | | | | | | |
| Testing option : Use whole annotation as reference set | | | | | | |
| **GO-ID** | **Description** | **p-value** | **corr p-value** | **cluster freq** | **total freq** | **Proteins listed by gene name** |
| 8135 | translation factor activity, RNA binding | 1.37E-02 | 2.93E-02 | 3/87 3.4% | 174/30449 0.5% | AT4G02930 AT3G62910 AT3G13920 |
| 3723 | RNA binding | 5.54E-01 | 6.09E-01 |  |  |  |
| 5198 | structural molecule activity | 2.40E-02 | 4.99E-02 | 5/87 5.7% | 571/30449 1.8% | AT4G01100 AT1G18080 AT5G62690 AT4G34620 AT5G09810 |
| 5840 | ribosome | 3.19E-03 | 8.20E-03 | 6/87 6.8% | 502/30449 1.6% | AT1G18080 AT3G02230 AT5G15650 AT4G34620 AT2G28000 AT5G02500 |
| 5730 | nucleolus | 1.38E-04 | 4.63E-04 | 7/87 8.0% | 394/30449 1.2% | AT1G13440 AT4G01850 AT2G30620 AT5G62690 AT3G13920 AT5G09810 AT5G02500 |
| 3676 | nucleic acid binding | 9.40E-01 | 9.53E-01 |  |  |  |
| 9607 | response to biotic stimulus | 1.22E-02 | 2.68E-02 | 9/87 10.3% | 1305/30449 4.2% | AT1G65930 AT4G37980 AT1G13440 AT4G08900 AT1G62660 AT3G12580 AT4G16260 AT3G57260 AT5G02500 |
| 9719 | response to endogenous stimulus | 9.12E-03 | 2.13E-02 | 11/87 12.6% | 1703/30449 5.5% | AT2G38910 AT1G18080 AT4G01850 AT1G70790 AT1G62660 AT5G45930 AT3G51850 AT1G77120 AT5G09810 AT1G21750 AT4G38970 |
| 9605 | response to external stimulus | 7.24E-03 | 1.74E-02 | 11/87 12.6% | 1649/30449 5.4% | AT1G65930 AT4G37980 AT1G13440 AT4G08900 AT3G03250 AT1G62660 AT3G12580 AT1G70730 AT4G16260 AT3G57260 AT5G02500 |
| 5794 | Golgi apparatus | 1.23E-04 | 4.30E-04 | 12/87 13.7% | 1171/30449 3.8% | AT2G33100 AT3G02230 AT5G56000 AT1G08660 AT5G03340 AT3G12580 AT2G14720 AT1G07750 AT5G15650 AT5G62690 AT1G55850 AT5G02500 |
| 6091 | generation of precursor metabolites and energy | 2.25E-11 | 3.47E-10 | 13/87 14.9% | 349/30449 1.1% | AT1G65930 AT4G01100 AT3G04120 AT1G13440 AT4G24620 AT3G22370 AT4G26910 AT1G70730 AT2G20420 AT4G11960 AT1G77120 AT4G38970 AT2G02050 |
| 5773 | vacuole | 5.31E-06 | 2.41E-05 | 15/87 17.2% | 1335/30449 4.3% | AT4G01100 AT3G04120 AT3G02230 AT5G18100 AT1G08660 AT1G62660 AT2G14720 AT3G59140 AT3G57260 AT5G56000 AT3G16480 AT3G12580 AT4G16260 AT1G21750 AT5G02500 |
| 9056 | catabolic process | 2.31E-06 | 1.11E-05 | 16/87 18.3% | 1417/30449 4.6% | AT5G19990 AT3G04120 AT5G42790 AT1G13440 AT5G47000 AT4G24620 AT2G40840 AT4G08900 AT5G23540 AT4G26910 AT1G12050 AT1G70730 AT4G29040 AT3G62760 AT5G58290 AT4G38970 |
| 7275 | multicellular organism development | 4.90E-03 | 1.22E-02 | 16/87 18.3% | 2769/30449 9.0% | AT3G04120 AT3G02230 AT3G08630 AT5G17310 AT3G03250 AT1G70730 AT5G15650 AT1G18080 AT4G29040 AT1G02050 AT3G11980 AT4G34620 AT5G09810 AT1G21750 AT4G30580 AT2G28000 |
| 30312 | external encapsulating structure | 3.24E-11 | 3.56E-10 | 17/87 19.5% | 743/30449 2.4% | AT3G02230 AT1G13440 AT4G02930 AT4G01850 AT5G15650 AT5G62690 AT3G57260 AT5G56000 AT2G19170 AT5G03340 AT3G12580 AT4G16260 AT5G58290 AT3G13920 AT5G09810 AT1G21750 AT5G02500 |
| 5618 | cell wall | 3.24E-11 | 3.56E-10 | 17/87 19.5% | 743/30449 2.4% | AT3G02230 AT1G13440 AT4G02930 AT4G01850 AT5G15650 AT5G62690 AT3G57260 AT5G56000 AT2G19170 AT5G03340 AT3G12580 AT4G16260 AT5G58290 AT3G13920 AT5G09810 AT1G21750 AT5G02500 |
| 5576 | extracellular region | 1.42E-03 | 4.05E-03 | 18/87 20.6% | 2933/30449 9.6% | AT1G65930 AT3G04120 AT5G47000 AT5G18100 AT4G01850 AT1G70730 AT3G57260 AT5G60710 AT5G56000 AT1G21230 AT2G19170 AT3G12580 AT1G08200 AT4G16260 AT3G13920 AT4G38970 AT2G28000 AT5G02500 |
| 5975 | carbohydrate metabolic process | 6.99E-10 | 5.98E-09 | 18/87 20.6% | 1038/30449 3.4% | AT2G33100 AT3G04120 AT3G02230 AT1G13440 AT4G24620 AT1G08660 AT2G40840 AT3G03250 AT1G62660 AT1G70730 AT5G15650 AT3G60750 AT1G55850 AT3G57260 AT1G32900 AT4G16260 AT4G38970 AT4G10260 |
| 16043 | cellular component organization | 3.49E-05 | 1.28E-04 | 19/87 21.8% | 2382/30449 7.8% | AT2G33100 AT3G02230 AT1G08660 AT2G17930 AT5G17310 AT3G03250 AT5G25880 AT5G15650 AT2G30620 AT1G55850 AT3G62910 AT3G16480 AT4G29040 AT1G02050 AT3G11980 AT1G08200 AT4G34620 AT5G09810 AT2G28000 |
| 16787 | hydrolase activity | 1.26E-03 | 3.72E-03 | 19/87 21.8% | 3150/30449 10.3% | AT5G19990 AT5G42790 AT5G47000 AT4G02930 AT4G08900 AT5G23540 AT1G62660 AT1G12050 AT3G59140 AT3G57260 AT3G16480 AT4G29040 AT2G19170 AT5G03340 AT5G45930 AT4G16260 AT5G58290 AT3G13920 AT3G28390 |
| 19538 | protein metabolic process | 1.10E-02 | 2.50E-02 | 20/87 22.9% | 4115/30449 13.5% | AT2G38910 AT4G01100 AT5G19990 AT5G42790 AT1G08660 AT4G02930 AT2G17930 AT5G23540 AT3G18060 AT3G62910 AT1G21230 AT3G16480 AT4G29040 AT2G19170 AT5G02790 AT3G12580 AT3G51850 AT4G34620 AT5G58290 AT3G13920 |
| 9628 | response to abiotic stimulus | 9.18E-08 | 5.44E-07 | 21/87 24.1% | 1943/30449 6.3% | AT1G65930 AT3G04120 AT3G02230 AT5G18100 AT3G22370 AT5G17310 AT3G03250 AT5G23540 AT1G70730 AT5G15650 AT5G62690 AT3G60750 AT3G57260 AT4G29040 AT3G12580 AT4G31970 AT4G16260 AT1G77120 AT5G09810 AT1G21750 AT5G02500 |
| 16740 | transferase activity | 1.51E-03 | 4.15E-03 | 21/87 24.1% | 3714/30449 12.1% | AT2G33100 AT2G38910 AT3G02230 AT1G08660 AT2G40840 AT4G01850 AT5G17310 AT3G03250 AT4G26910 AT3G60750 AT3G18060 AT1G55850 AT5G60710 AT1G32900 AT1G21230 AT5G02790 AT3G62760 AT1G02050 AT3G51850 AT4G30580 AT4G10260 |
| 5515 | protein binding | 2.51E-04 | 8.05E-04 | 22/87 25.2% | 3487/30449 11.4% | AT2G38910 AT5G19990 AT2G38700 AT3G02230 AT4G01850 AT5G15650 AT3G18060 AT3G55460 AT3G57260 AT1G18080 AT1G32900 AT5G56000 AT4G29040 AT5G03340 AT3G12580 AT1G02050 AT3G51850 AT1G77120 AT3G13920 AT5G09810 AT1G21750 AT5G02500 |
| 5886 | plasma membrane | 3.04E-05 | 1.17E-04 | 25/87 28.7% | 3733/30449 12.2% | AT2G33100 AT2G38910 AT5G19990 AT5G42790 AT1G13440 AT5G17310 AT2G14720 AT1G70730 AT3G59140 AT3G57260 AT1G18080 AT1G77120 AT3G28390 AT1G65930 AT3G04120 AT3G03250 AT5G62690 AT1G55850 AT5G60710 AT5G56000 AT1G21230 AT3G12580 AT3G51850 AT5G09810 AT5G02500 |
| 6950 | response to stress | 6.11E-07 | 3.14E-06 | 27/87 31.0% | 3439/30449 11.2% | AT5G19990 AT3G02230 AT1G13440 AT5G47000 AT5G18100 AT4G08900 AT5G17310 AT5G15650 AT3G60750 AT3G55460 AT3G57260 AT4G16260 AT1G77120 AT1G65930 AT3G04120 AT3G22370 AT3G03250 AT5G23540 AT5G62690 AT5G56000 AT5G36930 AT4G29040 AT3G12580 AT4G31970 AT5G09810 AT1G21750 AT5G02500 |
| 9058 | biosynthetic process | 4.76E-04 | 1.47E-03 | 29/87 33.3% | 5515/30449 18.1% | AT2G33100 AT3G02230 AT1G13440 AT4G01850 AT4G08900 AT1G70730 AT5G15650 AT3G60750 AT3G62910 AT4G37980 AT1G32900 AT1G08200 AT3G13920 AT2G02050 AT4G10260 AT4G01100 AT3G04120 AT2G38700 AT4G24620 AT1G08660 AT4G02930 AT1G55850 AT3G12580 AT1G02050 AT5G45930 AT4G31970 AT4G34620 AT4G30580 AT5G02500 |
| 5634 | nucleus | 4.72E-01 | 5.50E-01 |  |  |  |
| 166 | nucleotide binding | 2.92E-08 | 2.25E-07 | 29/87 33.3% | 3386/30449 11.1% | AT2G38910 AT5G19990 AT1G13440 AT4G01850 AT3G18060 AT3G55460 AT3G59140 AT1G18080 AT2G20420 AT5G03340 AT1G77120 AT3G13920 AT3G28390 AT4G10260 AT2G28000 AT1G65930 AT3G04120 AT2G38700 AT4G02930 AT5G25880 AT5G56000 AT5G36930 AT1G21230 AT4G29040 AT3G12580 AT5G45930 AT3G51850 AT5G09810 AT5G02500 |
| 9536 | plastid | 7.24E-08 | 5.07E-07 | 32/87 36.7% | 4211/30449 13.8% | AT3G08630 AT1G13440 AT5G18100 AT4G08900 AT2G07732 AT1G70730 AT3G60750 AT3G62910 AT3G57260 AT1G18080 AT1G32900 AT2G41520 AT4G38970 AT2G02050 AT4G10260 AT2G28000 AT1G65930 AT4G01100 AT3G04120 AT4G24620 AT2G40840 AT5G25880 AT5G56000 AT3G16480 AT4G11960 AT5G45930 AT4G31970 AT4G34620 AT5G09810 AT1G21750 AT4G30580 AT5G02500 |
| 5829 | cytosol | 1.69E-17 | 1.30E-15 | 33/87 37.9% | 1978/30449 6.4% | AT5G19990 AT3G02230 AT5G42790 AT1G13440 AT4G01850 AT5G17310 AT1G12050 AT1G70730 AT5G15650 AT3G18060 AT1G18080 AT5G02790 AT5G03340 AT1G07750 AT1G08200 AT5G58290 AT1G77120 AT3G13920 AT2G28000 AT1G65930 AT3G04120 AT2G38700 AT4G24620 AT2G40840 AT3G03250 AT5G23540 AT5G25880 AT5G56000 AT4G29040 AT3G62760 AT3G12580 AT5G09810 AT5G02500 |
| 16020 | membrane | 8.52E-08 | 5.44E-07 | 48/87 55.1% | 8510/30449 27.9% | AT2G33100 AT2G38910 AT5G19990 AT3G02230 AT5G42790 AT3G08630 AT1G13440 AT5G17310 AT4G26910 AT2G14720 AT1G70730 AT5G15650 AT3G60750 AT3G59140 AT3G57260 AT1G18080 AT4G16260 AT5G58290 AT1G77120 AT3G13920 AT3G28390 AT4G38970 AT2G02050 AT2G28000 AT1G65930 AT4G01100 AT3G04120 AT1G08660 AT3G22370 AT5G45530 AT3G03250 AT5G62690 AT1G55850 AT5G60710 AT5G56000 AT5G36930 AT1G21230 AT3G16480 AT4G11960 AT4G29040 AT2G19170 AT3G12580 AT3G51850 AT4G31970 AT5G09810 AT1G21750 AT4G30580 AT5G02500 |
| 5488 | binding | 1.95E-07 | 1.07E-06 | 58/87 66.6% | 11933/30449 39.1% | AT2G38910 AT5G19990 AT1G13440 AT5G18100 AT4G01850 AT2G07732 AT4G26910 AT3G55460 AT3G62910 AT3G59140 AT2G20420 AT1G70790 AT1G07750 AT1G08200 AT1G65930 AT3G04120 AT3G22370 AT4G02930 AT2G40840 AT5G60710 AT1G21230 AT4G29040 AT1G51580 AT3G12580 AT1G02050 AT5G45930 AT1G21750 AT3G02230 AT3G07200 AT5G47000 AT4G08900 AT2G14720 AT1G70730 AT5G15650 AT3G60750 AT3G18060 AT3G57260 AT4G37980 AT1G18080 AT1G32900 AT5G03340 AT1G77120 AT1G05990 AT3G13920 AT3G28390 AT4G10260 AT2G28000 AT2G38700 AT5G23540 AT5G25880 AT2G30620 AT5G56000 AT5G36930 AT3G16480 AT3G51850 AT4G31970 AT5G09810 AT5G02500 |
| 3824 | catalytic activity | 2.78E-13 | 6.71E-12 | 59/87 67.8% | 9079/30449 29.8% | AT2G33100 AT2G38910 AT5G19990 AT1G13440 AT5G18100 AT4G01850 AT4G26910 AT3G59140 AT2G20420 AT5G02790 AT1G08200 AT4G16260 AT4G38970 AT1G65930 AT3G04120 AT4G24620 AT3G22370 AT4G02930 AT2G40840 AT1G62660 AT1G55850 AT5G60710 AT1G21230 AT4G29040 AT3G62760 AT1G02050 AT5G45930 AT1G21750 AT4G30580 AT3G02230 AT5G42790 AT5G47000 AT4G08900 AT5G17310 AT1G12050 AT1G70730 AT5G15650 AT3G60750 AT3G18060 AT3G57260 AT4G37980 AT1G32900 AT5G03340 AT3G11980 AT5G58290 AT1G77120 AT3G13920 AT3G28390 AT2G02050 AT4G10260 AT2G38700 AT1G08660 AT3G03250 AT5G23540 AT5G25880 AT3G16480 AT2G19170 AT3G51850 AT4G31970 |
| 8152 | metabolic process | 1.33E-10 | 1.28E-09 | 62/87 71.2% | 11369/30449 37.3% | AT2G33100 AT2G38910 AT5G19990 AT1G13440 AT5G18100 AT2G17930 AT4G01850 AT4G26910 AT3G55460 AT3G62910 AT2G20420 AT5G02790 AT1G08200 AT4G16260 AT4G38970 AT1G65930 AT3G04120 AT4G24620 AT3G22370 AT4G02930 AT2G40840 AT1G62660 AT1G55850 AT1G21230 AT4G11960 AT4G29040 AT3G62760 AT3G12580 AT1G02050 AT5G45930 AT4G34620 AT4G30580 AT3G02230 AT5G42790 AT5G47000 AT4G08900 AT5G17310 AT1G12050 AT1G70730 AT5G15650 AT3G60750 AT3G18060 AT3G57260 AT4G37980 AT1G32900 AT3G11980 AT5G58290 AT1G77120 AT3G13920 AT2G02050 AT4G10260 AT4G01100 AT2G38700 AT1G08660 AT3G03250 AT5G23540 AT5G25880 AT3G16480 AT2G19170 AT3G51850 AT4G31970 AT5G02500 |
| 9987 | cellular process | 3.49E-13 | 6.71E-12 | 69/87 79.3% | 12509/30449 41.0% | AT2G33100 AT2G38910 AT5G19990 AT1G13440 AT5G18100 AT2G17930 AT4G01850 AT4G26910 AT3G55460 AT3G62910 AT2G20420 AT5G02790 AT1G70790 AT2G41520 AT1G08200 AT4G38970 AT1G65930 AT3G04120 AT4G24620 AT3G22370 AT4G02930 AT2G40840 AT1G62660 AT1G55850 AT1G21230 AT4G11960 AT4G29040 AT3G62760 AT3G12580 AT1G02050 AT5G45930 AT4G34620 AT1G21750 AT4G30580 AT3G02230 AT5G42790 AT5G47000 AT4G08900 AT5G17310 AT1G12050 AT1G70730 AT5G15650 AT3G60750 AT3G18060 AT4G37980 AT1G18080 AT1G32900 AT5G03340 AT3G11980 AT5G58290 AT1G77120 AT3G13920 AT2G02050 AT4G10260 AT2G28000 AT4G01100 AT2G38700 AT1G08660 AT3G03250 AT5G23540 AT5G25880 AT2G30620 AT5G56000 AT5G36930 AT3G16480 AT3G51850 AT4G31970 AT5G09810 AT5G02500 |
| 5737 | cytoplasm | 8.10E-14 | 3.12E-12 | 72/87 82.7% | 13344/30449 43.8% | AT2G33100 AT2G38910 AT5G19990 AT3G08630 AT1G13440 AT5G18100 AT4G01850 AT2G07732 AT4G26910 AT3G62910 AT3G59140 AT2G20420 AT5G02790 AT1G07750 AT2G41520 AT1G08200 AT4G16260 AT4G38970 AT1G65930 AT3G04120 AT4G24620 AT3G22370 AT4G02930 AT2G40840 AT1G62660 AT5G62690 AT1G55850 AT4G11960 AT4G29040 AT3G62760 AT3G12580 AT1G02050 AT5G45930 AT4G34620 AT1G21750 AT4G30580 AT3G02230 AT5G42790 AT4G08900 AT5G17310 AT1G12050 AT2G14720 AT1G70730 AT5G15650 AT3G60750 AT3G18060 AT3G57260 AT4G37980 AT1G18080 AT1G32900 AT5G03340 AT5G58290 AT1G77120 AT3G13920 AT3G07290 AT2G02050 AT4G10260 AT2G28000 AT4G01100 AT2G38700 AT1G08660 AT3G03250 AT5G23540 AT5G25880 AT2G30620 AT5G56000 AT5G36930 AT3G16480 AT3G51850 AT4G31970 AT5G09810 AT5G02500 |
| 5622 | intracellular | 7.29E-06 | 2.95E-05 | 77/87 88.5% | 20692/30449 67.9% | AT2G33100 AT2G38910 AT5G19990 AT3G08630 AT1G13440 AT5G18100 AT2G17930 AT4G01850 AT2G07732 AT4G26910 AT3G55460 AT3G62910 AT3G59140 AT2G20420 AT5G02790 AT1G07750 AT2G41520 AT1G08200 AT4G16260 AT4G38970 AT1G65930 AT3G04120 AT1G59690 AT4G24620 AT3G22370 AT4G02930 AT2G40840 AT1G62660 AT5G62690 AT1G55850 AT4G11960 AT4G29040 AT3G62760 AT3G12580 AT1G02050 AT5G45930 AT4G34620 AT1G21750 AT4G30580 AT3G02230 AT3G07200 AT5G42790 AT4G08900 AT5G17310 AT1G12050 AT2G14720 AT1G70730 AT5G15650 AT3G60750 AT3G18060 AT3G57260 AT4G37980 AT1G18080 AT1G32900 AT5G03340 AT5G58290 AT1G77120 AT3G13920 AT3G07290 AT2G02050 AT4G10260 AT2G28000 AT4G01100 AT2G38700 AT1G08660 AT3G03250 AT5G23540 AT5G25880 AT2G30620 AT2G32160 AT5G56000 AT5G36930 AT3G16480 AT3G51850 AT4G31970 AT5G09810 AT5G02500 |
| 5623 | cell | 7.06E-06 | 2.95E-05 | 81/87 93.1% | 22662/30449 74.4% | AT2G33100 AT2G38910 AT5G19990 AT3G08630 AT1G13440 AT5G18100 AT2G17930 AT4G01850 AT2G07732 AT4G26910 AT3G55460 AT3G62910 AT3G59140 AT2G20420 AT5G02790 AT1G07750 AT2G41520 AT1G08200 AT4G16260 AT4G38970 AT1G65930 AT3G04120 AT1G59690 AT4G24620 AT3G22370 AT4G02930 AT2G40840 AT1G62660 AT5G62690 AT1G55850 AT5G60710 AT1G21230 AT4G11960 AT4G29040 AT3G62760 AT3G12580 AT1G02050 AT5G45930 AT4G34620 AT1G21750 AT4G30580 AT3G02230 AT3G07200 AT5G42790 AT4G08900 AT5G17310 AT1G12050 AT2G14720 AT1G70730 AT5G15650 AT3G60750 AT3G18060 AT3G57260 AT4G37980 AT1G18080 AT1G32900 AT5G03340 AT5G58290 AT1G77120 AT3G13920 AT3G07290 AT3G28390 AT2G02050 AT4G10260 AT2G28000 AT4G01100 AT2G38700 AT1G08660 AT3G03250 AT5G23540 AT5G25880 AT2G30620 AT2G32160 AT5G56000 AT5G36930 AT3G16480 AT2G19170 AT3G51850 AT4G31970 AT5G09810 AT5G02500 |
| 8150 | biological_process | 8.24E-02 | 1.32E-01 |  |  |  |
| 5575 | cellular_component | 6.13E-01 | 6.55E-01 |  |  |  |
| 3674 | molecular_function | 2.41E-03 | 6.39E-03 | 85/87 97.7% | 27056/30449 88.8% | AT2G33100 AT2G38910 AT5G19990 AT3G08630 AT1G13440 AT5G18100 AT4G01850 AT2G07732 AT4G26910 AT3G55460 AT3G62910 AT3G59140 AT2G20420 AT5G02790 AT1G70790 AT1G07750 AT1G08200 AT4G16260 AT4G38970 AT1G65930 AT3G04120 AT1G59690 AT4G24620 AT3G22370 AT4G02930 AT2G40840 AT5G45530 AT1G62660 AT5G62690 AT1G55850 AT5G60710 AT1G21230 AT4G11960 AT4G29040 AT1G51580 AT3G62760 AT3G12580 AT1G02050 AT5G45930 AT4G34620 AT1G21750 AT4G30580 AT3G02230 AT3G07200 AT5G42790 AT5G47000 AT4G08900 AT5G17310 AT1G12050 AT2G14720 AT1G70730 AT5G15650 AT3G60750 AT3G18060 AT3G57260 AT4G37980 AT1G18080 AT1G32900 AT5G03340 AT3G11980 AT5G58290 AT1G77120 AT1G05990 AT3G13920 AT3G07290 AT3G28390 AT2G02050 AT4G10260 AT2G28000 AT4G01100 AT2G38700 AT1G08660 AT3G03250 AT5G23540 AT5G25880 AT2G30620 AT2G32160 AT5G56000 AT5G36930 AT3G16480 AT2G19170 AT3G51850 AT4G31970 AT5G09810 AT5G02500 |
