# Supplementary material for: Chemical hybridizing agent SQ-1-induced male sterility in Triticum aestivum L.: a comparative analysis of the anther proteome
Source: BMC Plant Biol. 2018 Jan 5;18:7. doi: 10.1186/s12870-017-1225-x (PMC5755283; doi:10.1186/s12870-017-1225-x)
Supplement: Supplementary file 10 — Results of Sugar metabolism related enzyme activity and qRT-PCR. (DOCX 155 kb) [file 12870_2017_1225_MOESM10_ESM.docx]

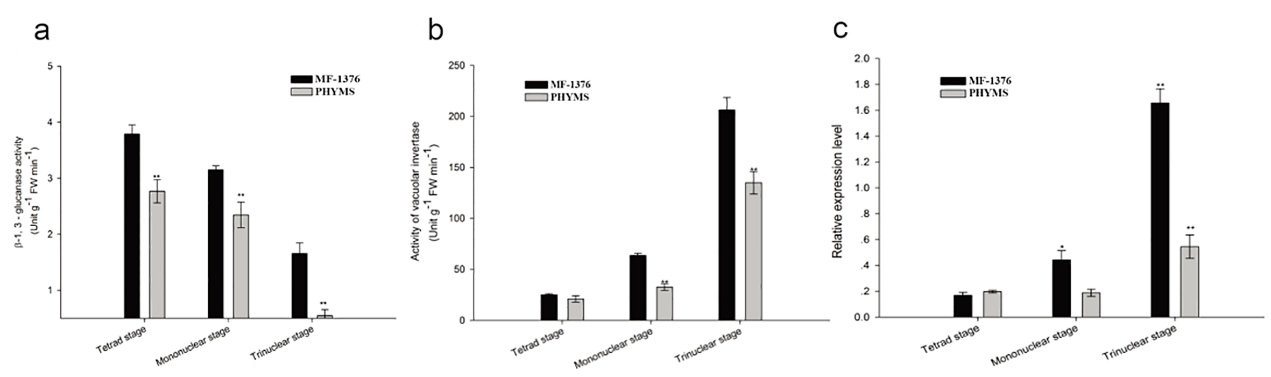


**Figure S6. Results of Sugar metabolism related enzyme activity and qRT-PCR.** The β-1, 3-glucanase activity (a) and vacuolar invertase activity (b) in anthers of PHYMS at tetrad, mononuclear and trinucleate stage compared with the corresponding of MF-1376. Quantitative real-time RT-PCR using SYBR Green assays for quantitative analysis of vacuolar invertase gene of mRNA expression levels in anthers at three stages between MF-1376 and PHYMS (c). Data are the mean±SD from three replications. *, ** = significantly different from the control (tetrad anther of MF-1376) at p < 0.05 and p < 0.01, respectively.
